# Laveran décalé

**DOI:** 10.48327/mtsi.v3i3.2023.406

**Published:** 2023-08-01

**Authors:** Pierre L. GOOSSENS

**Affiliations:** 1 Unité Yersinia, Institut Pasteur, 25 Rue Du Docteur Roux 75015 Paris, France

**Keywords:** Alphonse Laveran, Marie Laveran, Marie Phisalix, Prix Nobel, Histoire de la médecine, Archives, France, Alphonse Laveran, Marie Laveran, Marie Phisalix, Nobel Prize, History of medicine, Archives, France

## Abstract

Charles Louis Alphonse Laveran : 18 juin 1845 – 18 mai 1922. Premier prix Nobel de médecine français, « en reconnaissance de ses travaux sur le rôle joué par les protozoaires dans l'apparition des maladies ». Cent ans après sa disparition ne restent plus que des traces écrites de ses travaux et de sa vie. Les témoins de cette période ne sont plus. Alphonse Laveran est devenu un « objet » d'Histoire.

Il fut un acteur profondément intégré dans une période historique mouvementée, marquée par des crises de changement de régime (Monarchie/Empire/République), par des événements militaires (expansion coloniale française en Afrique du Nord depuis 1830, guerres de 1870 et 1914-1918) et leurs conséquences (impact médical des infections dans l'empire colonial et lors des conflits armés, affaire Dreyfus entre autres), par l'avènement de la « microbiologie » pasteurienne et le déchiffrement des causes et modes de transmission des maladies infectieuses. Acteur à la lisière des mondes militaire et civil, avec leurs propres visions des buts et objectifs à suivre, parfois incompatibles, Alphonse Laveran vécut ces bouleversements dans une société en plein changement, dans son milieu familial et scientifique.

Les sources primaires à notre disposition pour aborder ce personnage scientifique, et cet homme, sont paradoxalement à la fois abondantes et « pauvres » pour nous, curieux du XXI^e^ siècle. Ses publications scientifiques et nombre de ses interventions dans les différentes académies, comités et réunions sont pour la plupart publiques, accessibles et nous donnent une vision du professionnel de la recherche scientifique et médicale en action pour présenter et convaincre de ses idées et fulgurances théoriques et pratiques. Les écrits de ses contemporains, qu'ils soient publics ou privés éclairent – et déforment ? - de multiples facettes du personnage. A l'opposé, peu de sources persistent quant à l'homme et sa vision de la vie, de sa vie et celle de sa famille et de ses proches.

Nous nous appuierons sur les archives qui ont été conservées, en particulier par les structures et corps l'ayant accueilli au cours de sa carrière militaire et civile, ainsi que par son épouse Marie Laveran et sa collègue Marie Phisalix, une des premières docteures en médecine en France et herpétologiste de renom; ces deux personnalités féminines ont préservé et contribué à son souvenir. Nous tenterons d'approcher l'homme derrière le scientifique, tel que nous pouvons l'imaginer à travers les traces qui nous restent.

## Abstract (en)

Charles Louis Alphonse Laveran - 18 June 1845 - 18 May 1922: first French Nobel Prize in Medicine, “in recognition of his work on the role played by protozoa in causing diseases”. One hundred years after his death, only written records remain of his work and life. The witnesses to this period are no more. Alphonse Laveran has become an “object” of history.

He was deeply involved in a turbulent historical period, marked by crises of regime change (Monarchy/Empire/Republic), military events (French colonial expansion in North Africa from 1830, the wars of 1870 and 1914-1918) and their consequences (the medical impact of infections in the colonial empire and during armed conflicts, the Dreyfus affair, among others), the advent of Pasteurian “microbiology” and the deciphering of the causes and modes of transmission of infectious diseases. A player on the edge of the military and civilian worlds, with their own, sometimes incompatible, visions of the aims and objectives to be pursued, Alphonse Laveran lived through these upheavals in a society in the throes of change, in his family and scientific environment.

Paradoxically, the primary sources available to us for learning about this scientist and man are both abundant and “scarce” for us in the 21st century. His scientific publications and many of his speeches at various academies, committees and meetings are for the most part public and accessible, giving us a vision of a professional in scientific and medical research in action, presenting and convincing people of his ideas and theoretical and practical insights. The writings of his contemporaries, both public and private, shed light on - distort? - the man's many facets. On the other hand, there are few surviving sources on the man and his vision of life, his life and that of his family and friends.

We will rely on the archives that have been preserved, in particular by the organisations that welcomed him during his military and civilian career, as well as by his wife Marie Laveran and his colleague Marie Phisalix, one of the first doctors of medicine in France and a renowned herpetologist. These two female figures have preserved and contributed to his memory. Let's take a closer look at the man behind the scientist, as we can imagine him through the traces that remain.

## Introduction

« L'autonomie de la vie se révèle imprévisiblement, le tout est de saisir le fait apparemment contradictoire à l'observation commune et de l'intégrer dans la connaissance au lieu de le rejeter comme une aberration de la nature […] car en expérimentation biologique, l'essentiel n'est pas de poursuivre toujours le but que l'on cherchait, mais de voir aussi ce qu'on n'y cherchait pas [8]. »

Le 6 novembre 1880, date de « La Découverte » – comme Alphonse Laveran baptise luimême ce moment où tout bascule – marque sa description du parasite du paludisme vu au microscope. Évènement fondateur de sa carrière, tant par sa reconnaissance que par ses détracteurs, il est « mis en scène » dans ses textes fondateurs et repris régulièrement depuis; il entre ainsi dans les mythes de découverte comme la formule de Kékulé, Descartes et son poêle, Einstein et un troupeau de vaches, Newton et la pomme, Kary B. Mullis et la PCR… La suite est connue : premier prix Nobel de médecine français, « en reconnaissance de ses travaux sur le rôle joué par les protozoaires dans l'apparition des maladies ». Cent ans après sa disparition ne restent plus que des traces écrites de ses travaux et de sa vie. Les témoins de cette période ne sont plus. Alphonse Laveran est devenu un « objet » d'Histoire.

Il fut un acteur profondément intégré dans une période historique mouvementée, marquée par des crises de changement de régime (Monarchie, Empire, République), des évènements militaires (expansion coloniale française en Afrique du Nord depuis 1830, guerres de 1870 et 1914-1918) et leurs conséquences (impact médical des maladies tropicales dans l'empire colonial, et infections lors des conflits armés, affaire Dreyfus entre autres), l'avènement de la « microbiologie » pasteurienne et le déchiffrement des causes et modes de transmission des maladies infectieuses. Acteur à la lisière des mondes militaire et civil, avec leurs propres visions des buts et objectifs à suivre, parfois incompatibles, Alphonse Laveran vécut aussi ces bouleversements dans son milieu familial et scientifique.

Les sources primaires à notre disposition pour aborder ce personnage scientifique, et cet homme, sont paradoxalement à la fois abondantes et pauvres pour nous, curieux du xxi^e^ siècle. Ses publications scientifiques et nombre de ses interventions dans les différentes académies, comités et réunions sont pour la plupart publiques, accessibles et nous donnent une vision du professionnel de la recherche scientifique et médicale en action pour présenter et convaincre de ses idées et fulgurances théoriques et pratiques^1^1La plateforme PaJ@Mo du CeRIS à l'Institut Pasteur est l'une des ressources disponibles (https://bibnum.pasteur.fr/app/photopro.sk/pasteur/?#biblio/50056), ainsi que la photothèque (https://phototheque.pasteur.fr/fr/asset/fullTextSearch/search/laveran).. Les écrits de ses contemporains, qu'ils soient publics ou privés éclairent – et déforment ? – de multiples facettes du personnage. À l'opposé, peu de sources persistent quant à l'homme et sa vision de la vie, de sa vie et celle de sa famille et de ses proches.

Je me suis appuyé sur les archives qui ont été conservées, en particulier par les structures et corps l'ayant accueilli au cours de sa carrière militaire et civile, ainsi que par son épouse Marie Laveran et sa collègue Marie Phisalix^2^2Marie Phisalix (1861-1946), épouse de Césaire Phisalix inventeur du sérum antivenimeux, était normalienne et l'une des premières docteures en médecine française (1900), herpétologiste de renom. – deux personnalités féminines qui ont préservé et contribué à son souvenir. Tentons d'approcher l'homme derrière le scientifique, tel que nous pouvons l'imaginer à travers les traces qui nous restent; nous posant la question de l'actualité de cette approche dans nos environnements professionnels; nous interrogeant sur quand et comment pressentir ce qui devrait être préservé pour l'Histoire future à écrire, dans un monde de plus en plus riche en production d'informations parfois de plus en plus volatiles.

## PARCOURS D'UNE VIE

Charles Louis Alphonse Laveran naît le 18 juin 1845 à Paris, rue de l'Est dans le 12^e^ arrondissement de l’époque, le 6^e^ actuel, tout près du Val-de-Grâce où était affecté son père, Louis Théodore Laveran. Cette rue a disparu lors du percement du boulevard Saint Michel. Sa venue au monde a été précédée par celle de sa soeur, Caroline, née en 1842 à Metz lors de la précédente affectation de son père. Il est baptisé le 6 novembre 1845 en la paroisse Saint-Sulpice, avec comme parrain et marraine, Alphonse et Élisa de Lignières^3^3Registre des actes de Baptême (AIP (Archives, Musée et Bibliothèque de l'Institut Pasteur) LAV.4)..

Le parcours de vie d'Alphonse Laveran peut être abordé de plusieurs points de vue.

Tout d'abord, *via* les évènements de sa carrière : une période « militaire » d'environ 17 ans, avec des études de médecine à l’École impériale de Strasbourg; « La Découverte » le 6 novembre 1880; la déchirure en 1894-1896 aboutissant à sa démission de l'institution militaire et au basculement dans sa période « civile » qui dura 26 ans, marquée par son entrée à l'Institut Pasteur [18]; le prix Nobel en 1907 [3]; ses appartenances aux différentes Académies [4] et son décès le 18 mai 1922 à l’âge de 77 ans.

Ou bien *via* le contexte historique : Alphonse Laveran a vécu dans une société en pleine mutation et historiquement très riche en évènements. Il est né sous Louis-Philippe, roi des Français. Il a 3 ans au moment de la révolution de 1848 et de l'instauration de la Seconde République avec le prince-président Louis Napoléon Bonaparte. Au moment du coup d’État du 2 décembre 1851, il se trouve en Algérie où son père Louis Théodore Laveran est affecté; ce qui, *a posteriori*, pourrait être considéré comme « *a blessing in disguise* », lui évitant ainsi d'avoir à se positionner vis-à-vis du coup d’État.

Alphonse Laveran, à partir de l’âge de 10 ans, vécut ainsi pendant toute la période du Second Empire. Il poursuivit ses études et sa formation sous les institutions impériales, dont la fameuse École impériale du Service de santé militaire à Strasbourg [15]. Il subit de plein fouet la guerre franco-prussienne de 1870, il a alors 25 ans, en tant que médecin militaire, pendant le siège de Metz en 1870. De retour à Paris le 22 mars 1871, il est confronté directement aux évènements de la Commune de Paris dans son affectation à l'hôpital Saint-Martin dans le 10^e^ arrondissement établi dans l'ancien couvent des Récollets. La Troisième République va enfin accompagner le restant de sa longue vie. Cette période ne fut pas une longue période tranquille politiquement, aboutissant en particulier à la Première Guerre mondiale (il a alors 70 ans).

Un des évènements majeurs de cette période fut l'Affaire, comme a été dénommé ce bouleversement de la société française, l'affaire Dreyfus qui divisa profondément la France. Or cette affaire débuta en 1894, année de la fin du professorat d'Alphonse Laveran au Val-de-Grâce (1884-1894). Picquart découvrit les erreurs et irrégularités du dossier en 1896 et l'Affaire éclata au grand jour fin 1897 dans les journaux (*Le Temps*, *Le Figaro*); ainsi se trouvèrent réunis tous les engrenages aboutissant à cette crise majeure de la société française. Quelle fut la position de Laveran sur cette affaire ? Quelles ont pu en être les conséquences sur cette période de sa carrière où se jouaient ses réaffectations et son positionnement vis-à-vis de l'institution de la médecine militaire et de ses responsabilités ? Il est intéressant de constater qu'il n'existe actuellement aucune information connue dans les archives sur sa position, sauf… une mention – sibylline – par Félix Mesnil qui a bien connu Alphonse Laveran pour l'avoir côtoyé pendant de nombreuses années à l'Institut Pasteur. En effet, dans le discours officiel qu'il fit à Constantine le 23 mai 1930, lors de la « cérémonie commémorative de la découverte par Alphonse Laveran de l'hématozoaire du paludisme », il énonce : « Il avait un patriotisme concentré et un sentiment foncier de justice qu'il sut exprimer autour de lui dans les circonstances qui ont divisé la France il y a 30 ans. Jamais il ne sacrifia à son indépendance d'esprit [14]. »

Dans un Institut où Émile Duclaux s'est impliqué dès la première heure lorsque le vice-président du Sénat Auguste Scheurer-Kestner vint le voir en 1897, et où Félix Mesnil dans une lettre à Paul Simond du 12 avril 1899 décrit ainsi Émile Roux : « En ce moment, il est tout à l'affaire Dreyfus; c'est le plus dreyfusard de la bande, il ne s'intéresse plus qu’à cela^4^4AIP SIM.4.. » On a du mal à imaginer qu'Alphonse Laveran n'ait pas pris position alors que les témoignages de ceux qui l'ont connu au cours de sa vie soulignent son honnêteté foncière, sa droiture. Cependant, sa formation militaire et son respect pour l'Institution militaire ne l'empêchaient probablement pas d'exprimer en privé son opinion et ne lui ôtaient sûrement pas son franc-parler pour défendre cette même institution et refuser ses errements. Aucun document pour l'instant ne montre que cette affaire ait eu un rôle direct dans les refus d'affectations de sa hiérarchie dans des postes qui lui auraient permis de poursuivre sa carrière de médecin chercheur. Cependant, l'ambiance régnant dans les sphères décisionnelles, tant au niveau militaire que politique, ne pouvait probablement pas permettre de considérer sa demande comme recevable, noyée dans les tentatives de circonscrire le scandale qui secouait l'Institution militaire. N'oublions pas que c'est le courrier officiel du général Billot, ministre de la Guerre en 1897, qui provoqua la démission d'Alphonse Laveran, Billot qui faisait à l’époque tout ce qu'il pouvait pour étouffer le scandale. À moins de dénicher des documents originaux, nous ne connaîtrons sans doute jamais l'impact que cette période eut sur cette déchirure dans la carrière de Laveran. Cela laisse de la place à l'imagination…

## ALPHONSE LAVERAN HOMME DE COMMÉMORATION…

En se promenant dans la bibliographie autour d'Alphonse Laveran, on s'aperçoit qu'une grande partie des publications à son sujet relèvent des commémorations qui ont été effectuées à différents moments pour célébrer les épisodes marquants de sa vie.

Le 16 juin 1915 à l'Institut Pasteur, le jubilé pour ses 70 ans; en mai 1930 à Alger et Constantine, et le 6 novembre 1930 au Valde-Grâce, les 50 ans de « La Découverte »; les 25 ans de la Société de pathologie exotique les 8 et 9 février 1933; le 12 juillet 1945 au grand amphithéâtre de la Sorbonne, le centenaire de sa naissance; les 18 et 19 novembre 1958 dans le grand amphithéâtre de l'Institut Pasteur, les 50 ans de la fondation de la Société de pathologie exotique (SPE); en 1980 à Strasbourg, le centenaire de « La Découverte »; en 2007 à l'Académie de médecine, le centenaire du prix Nobel; et en cette année 2022, le centenaire de sa disparition… Commémorer, c'est rappeler à la mémoire. Avec le temps qui s’écoule inexorablement, ces commémorations permettent de transmettre à nos plus jeunes ce que fut une vie de scientifique et de médecin, marquée par la curiosité et l'indépendance, et ce qu'elle peut nous apporter dans notre approche de recherche dans la société actuelle, militaire ou civile. Comme nous verrons plus loin, Alphonse Laveran s'est efforcé tout au cours de sa vie à maîtriser son image; nul doute qu'il aurait apprécié le déroulement de cette mémoire collective à son égard.

## ALPHONSE LAVERAN ET SON PÈRE (Fig. 1)

**Figure 1:**
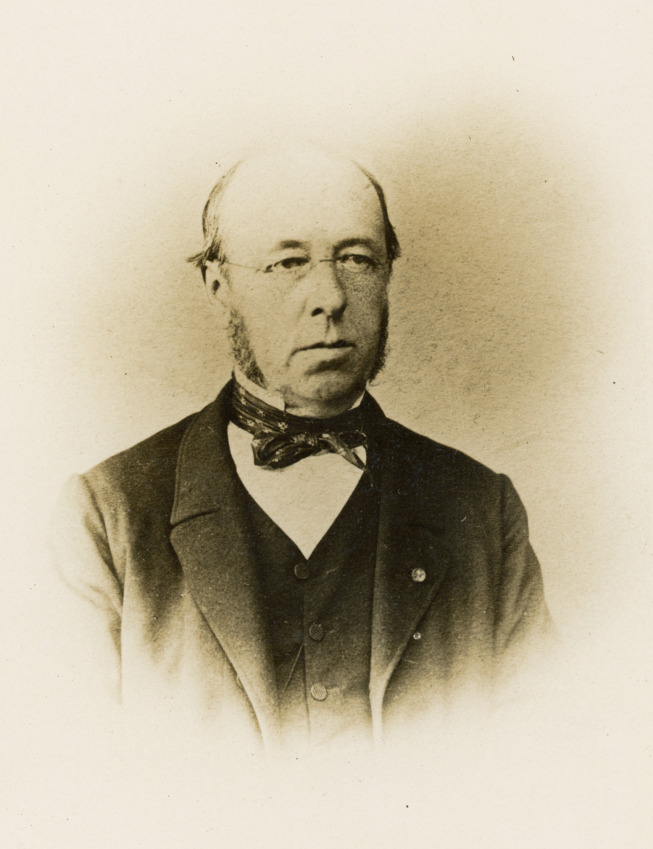
Louis Théodore Laveran (1812-1879), père d'Alphonse Laveran. Professeur puis directeur de l’École de médecine militaire du Val-de-Grâce (crédit photo : Institut Pasteur/Archives – Fonds Alphonse Laveran) Louis Théodore Laveran (1812-1879), father of Alphonse Laveran. Professor and later director of the Val-de-Grâce School of Military Medicine (photo credit: Institut Pasteur/Archives - Fonds Alphonse Laveran)

Replongeons-nous dans Alphonse Laveran et sa vie. Un document manuscrit curieux, intitulé « à la mémoire du Docteur Laveran, médecin inspecteur de l'Armée » apporte un éclairage particulier^5^5AIP-LAV.2.. Ce document fut rédigé par Charles François Alexandre Perron (1824-1892), probablement en 1891, « dans la dernière année de sa vie » comme le mentionne son fils le 17 février 1908 dans une lettre à Alphonse Laveran. Il décrivait les souvenirs de ses études à l’école militaire de Metz et de ses interactions avec Louis Théodore Laveran, père d'Alphonse. Louis Théodore Laveran fut affecté à Metz de février 1841 à novembre 1843 et succéda à Michel Lévy comme directeur.

« Il n'avait pas [au même degré que lui] (citant Michel Lévy) le goût de la pose ni de l'apparât [sic]; il ne cherchait pas à faire du prestige. En digne Flamand qu'il était, il avait une grande simplicité de manière et de tenue. Il était réservé, presque froid et peu causeur, quoique très affable toujours avec son personnel.

On le voyait rarement en tenue militaire, le plumet ne lui allait pas. On aurait même dit qu'il cherchait à fuir les honneurs.

Méditatif et rêveur, avec cela très myope, il ne voyait bien qu’à l'aide de son microscope, qu'il savait admirablement manier; car Laveran était aussi savant que modeste. »

En parcourant ce document, nous ressentons une étrange sensation, tellement cette description de Louis Théodore Laveran pourrait s'appliquer à Alphonse Laveran, ainsi que ceux qui l'ont connu nous l'ont transmis. Il est troublant de voir combien le père et le fils se ressemblaient dans ces descriptions par leurs contemporains, et pose de façon curieuse le lien filial existant entre Alphonse et Louis Théodore. Outre certains parallèles entre leurs carrières, tous deux professeurs agrégés du Val-de-Grâce et spécialistes des maladies infectieuses, Alphonse Laveran eut cependant le privilège de vivre au moment de l’éclosion de la microbiologie pasteurienne et de ses concepts. Contrairement à son père qui fut directeur de l’École du Val-de-Grâce à la fin de sa carrière (1872-1875), la carrière militaire d'Alphonse Laveran fut écourtée, malheureusement pour l'institution militaire et au moins temporairement pour lui-même, le temps qu'il réoriente sa carrière vers le civil, mais fort heureusement pour l'Institut Pasteur. Il apporta ainsi son expertise dans le monde non bactérien qui émergeait alors, celui des « protozoaires pathogènes^6^6Lettre d'Alphonse Laveran à Émile Roux pour le don de 100 000 Fr à l'Institut Pasteur à la suite de l'attribution du prix Nobel – MSSA (Musée et Bibliothèque du Service de santé des armées Val-de-Grâce) C1043_dos14. » avec leurs spécificités, eucaryotes *versus* procaryotes, conditions de culture encore inconnues – rappelons que les cultures de tissus et de cellules eucaryotes ne sont intervenues que plus tardivement, après 1902 (Gottlieb Haberlandt, Alexis Carrel entre autres pionniers), cycles épidémiologiques complexes à déchiffrer –, les postulats de Koch ne pouvant encore être appliqués aisément pour ce nouveau monde de pathogènes. Cependant, les travaux de Pasteur sur la rage montrent qu'il était possible d'adresser et explorer des types d'agents pathogènes atypiques, filtrables, cultivables uniquement *in vivo* à l’époque [2]; rappelons que les virus en tant qu'entité biologique ne furent définis précisément que dans les années 1950 [12].

Louis Théodore Laveran, professeur au Valde-Grâce de 1856 à 1867, directeur de l’École à partir de 1872, à la retraite depuis 1878^7^7MSSA C1042. a vécu au 127 boulevard Saint Michel, tout près du Val-de-Grâce où une partie importante de sa carrière s'est déroulée; il y est décédé le 20 août 1879^8^8AP (Archives de Paris) DQ7 11403 succession Louis Théodore Laveran.. Alphonse Laveran était alors affecté à Constantine en Algérie depuis septembre 1878. À cette époque, les moyens de communication ne présentaient pas l'immédiateté d'aujourd'hui; quant aux moyens de transport, ils ne permettaient pas les liaisons rapides auxquelles nous sommes habitués. Les recherches dans les archives militaires n'ont pas permis de retrouver la mention d'une permission de voyage pour la métropole, voire d'une simple demande. Alphonse Laveran n'a donc pas assisté aux derniers moments de son père, ni à son inhumation. Une cérémonie fut tenue en l’église Saint-Jacques du Haut Pas^9^9Mémorial de la Loire et de la Haute-Loire, 22 août 1879 – RetroNews. à proximité de la demeure paternelle. En son absence, c'est sa soeur Caroline qui s'est occupée des démarches, tel que cela peut se lire dans l'acte de succession signé par ellemême; elle est mentionnée comme vivant au domicile de ses parents dans les actes de décès et succession^10^10AP 05 V4E 3073 & DQ7 12984.. Des frais d'inhumation de 880 Fr sont reportés dans les registres de pompes funèbres^11^11AP 2484 W1., couvrant probablement le transfert et l'inhumation au cimetière de Dunkerque, sa ville natale, où se trouvait une concession familiale. Dans les *Mémoires de la société Dunkerquoise* [6], on peut lire en effet page 364:

« TOMBEAU DE FAMILLE CI-GISENT:

Suivent : 1° les noms de six membres de la famille LAVERAN; 2° […]

3° du Dr Louis LAVERAN, médecin-inspecteur des armées, commandeur de la Légion d'honneur;

4° de Mlle Louise LAVERAN, institutrice, Officier d'académie, décédée le 4 juin 1894. »

**Figure 2:**
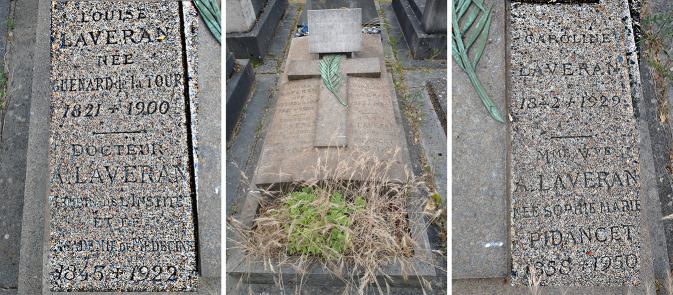
Tombe familiale d'Alphonse Laveran au cimetière du Montparnasse, Paris 15^e^, au 16 juin 2023. Photo retouchée accentuée pour les parties gravées. Se distinguent une palme ainsi que la plaque apposée par la SPE en 1980 à l'occasion du centenaire de La Découverte (crédit photo : Pierre L. Goossens) Alphonse Laveran's family grave in the Montparnasse cemetery, 75015 Paris, 16 June 2023. Photo retouched and enhanced for the engraved parts. Near the palm is the plaque affixed by the SPE in 1980 to mark the centenary of “the Discovery” (photo credit: Pierre L. Goossens)

Lors du décès de Marie Guénard de la Tour, veuve de Louis Théodore Laveran, le 18 décembre 1900, Alphonse Laveran prend la décision d'achat d'une concession perpétuelle au cimetière du Montparnasse à Paris, non loin de son domicile. Il s'y fera inhumer en 1922, puis sa soeur Caroline l'y rejoindra en 1929, et enfin Marie Laveran son épouse en 1950^12^12Cette sépulture se trouve localisée dans la 17e division, 10e ligne Est, n° 2 Sud, 258-1900. Aymé Camelin, dans une lettre du 18 juin 1980 (AIP SPE.B2) écrit : « Et enfin avec l'aide du Souvenir Français, dont j'ai l'accord, [je voudrais] faire rénover les tombes de Laveran et Maillot au cimetière du Montparnasse. Je me suis posé la question de savoir si Laveran n'avait pas acheté la concession avec l'arrière-pensée de faire reposer sa mère puis sa femme et enfin lui-même à 10 mètres de la tombe de Maillot. Ce que je ne sais pas, c'est s'il existe des descendants collatéraux qui seraient connus de l'Institut Pasteur. Peut-être le savez-vous ? » Pour le centenaire de « La Découverte » en 1980, la tombe a été rénovée, fleurie et une plaque apposée par la SPE. Dans sa lettre du 29 juin 1980 en réponse à son correspondant (AIP SPE. B2), Aymé Camelin écrit : « Je suis content que vous ayez fleuri et rénové la tombe de Laveran au nom de la Société de pathologie exotique. J'ai des photographies des tombes de Laveran et Maillot, que je connais bien, m'y rendant en pèlerinage chaque année. La dernière fois ce fut le 20 avril 1980, avant que vous n'ayez fait rénover la tombe de Laveran. À un prochain voyage à Paris je ferai nettoyer la tombe de Maillot et entretenir celle de Laveran (par le Souvenir Français). » Certaines mentions dans ces lettres font penser que le destinataire pourrait être André Dodin. En 2023, 43 ans plus tard, le temps a malheureusement fait son oeuvre… À l'occasion du centenaire de la disparition de Laveran, le CA du 23 novembre 2022 de la SFMTSI (ex-SPE) a adopté à l'unanimité le principe d'une démarche de la société auprès de la mairie de Paris pour obtenir le droit d'entretenir la sépulture Laveran en l'absence d'héritiers connus (e-mail de Jean-Paul Boutin du 25 juin 2023). (Fig. 2).

Ceci explique dans une certaine mesure pourquoi Louis Théodore Laveran et Marie Guénard de la Tour reposent séparément. Cependant, un esprit curieux pourrait se poser la question des raisons du non-transfert de Louis Théodore Laveran auprès de son épouse; décision d'Alphonse Laveran, en concertation avec sa soeur Caroline qu'il chérissait ? Un pan de vie inconnu… N'oublions pas que ces évènements se déroulent à la charnière des xix^e^ et xx^e^ siècles où ces choix familiaux reposaient sur des normes sociales à respecter. Enfin, Alphonse Laveran a conservé toute sa vie un portrait de son père, ainsi mentionné dans son testament : « Je lègue au Musée de Dunkerque le portrait de mon père par Horace Vernet, qui se trouve dans mon cabinet de travail, ainsi que les médailles décernées à mon père, qui sont dans le coffre-fort^13^13AN (Archives nationales) MC/ET/L/NC/125 et 129.. » Le musée des beaux-arts de Dunkerque a fermé au 1^er^ avril 2015; est-ce à ce musée que le don de ce portrait a été fait ? Et a-t-il été accepté ? Comment se trouve représenté son père ?

Encore une piste de recherche… mais le fait qu'Alphonse Laveran l'ait conservé toute sa vie, 22 ans après le décès de sa mère, suggère l'importance qu'il lui portait.

## ALPHONSE LAVERAN EN DEHORS DU LABORATOIRE

Comment approcher Alphonse Laveran en dehors du laboratoire et de son travail de scientifique ? Pour cela, nous devons nous reposer sur les documents émanant des contemporains qui l'ont côtoyé. Alphonse Laveran a laissé nombre d’écrits et interventions dans les différentes structures au sein desquelles il a oeuvré, mais il est resté fort discret, peu disert, très en retrait sur la vie de tous les jours et ses interactions publiques ou privées. Il serait néanmoins intéressant de chercher au sein de ses interventions publiques ce qui représenterait une empreinte, une trace, une imprégnation de ses positions philosophiques personnelles, sur la vie en général; un chantier en perspective…

En recherchant dans les écrits de ses contemporains, parfois certaines réflexions se font jour. Le dilemme actuel est de percevoir ce qui peut relever de l'hagiographie ou de la description d'un ressenti « réel » de l'interlocuteur, pouvant ainsi permettre d'approcher Alphonse Laveran.

**Figure 3:**
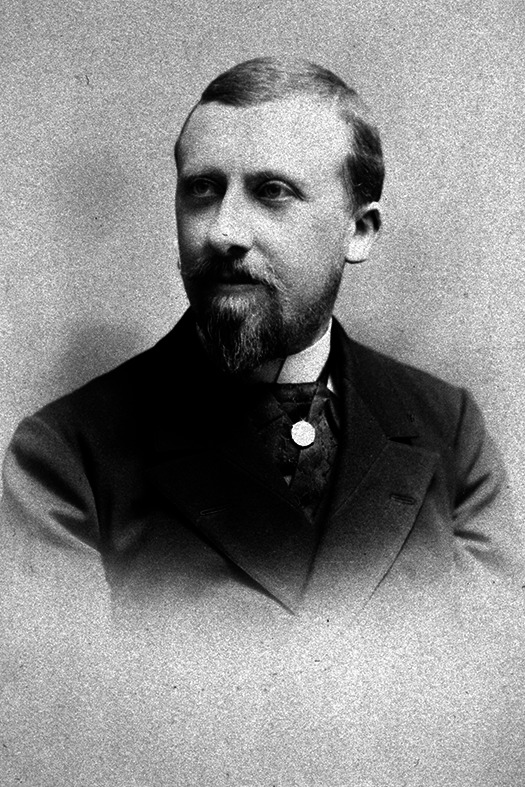
Félix Mesnil (1868-1938) vers 1900-1910. Zoologiste entré à l'Institut Pasteur en 1892 comme préparateur de Louis Pasteur, élève de Metchnikoff, il devint chef du service de microbiologie coloniale en 1907. Il partagea pendant de nombreuses années le même laboratoire avec Alphonse Laveran où ils développèrent les recherches sur les protozoaires pathogènes. Il créa en 1903 le *Bulletin de l'Institut Pasteur*. Quand fut fondée la SPE le 22 janvier 1908, Félix Mesnil fut avec Émile Marchoux le premier secrétaire général, avant d'en devenir le 3^e^ président en 1924 (crédit photo : Institut Pasteur/Musée Pasteur) Félix Mesnil (1868-1938) circa 1900-1910. A zoologist who joined the Institut Pasteur in 1892 as Louis Pasteur's assistant and was a pupil of Metchnikoff, he became head of the colonial microbiology department in 1907. For many years he shared the same laboratory with Alphonse Laveran, where they developed research into pathogenic protozoa. In 1903, he founded the Bulletin de l'Institut Pasteur. When the SPE was founded on 22 January 1908, Félix Mesnil, along with Émile Marchoux, was its first secretary general, before becoming its 3rd president in 1924 (photo credit: Institut Pasteur/Musée Pasteur)

Tout un travail de recherche dans les documents archivés serait nécessaire pour tenter de discerner l'homme qu'il était, scientifique, médecin, militaire et privé. Ne pouvant être exhaustif, et en gardant à l'esprit que les touches du portrait qui vont apparaître ci-après ne représenteront qu'une facette du personnage, donc forcément biaisée, nous déroulerons quelques bribes glanées dans les courriers, en particulier dans ceux de Félix Mesnil (Fig. 3). En effet, en 1897, lors de sa démission du Service de santé et de son accueil à l'Institut Pasteur, « [Laveran] partagea pendant plusieurs années avec M. Mesnil une unique chambre de travail, où s’établit bientôt leur fructueuse collaboration [19]. »

Félix Mesnil et Alphonse Laveran ont participé ensemble au développement de la protozoologie, et cela ne se passait pas toujours sans tension comme cela peut se percevoir dans ces quelques échanges épistolaires entre Félix Mesnil et Charles Nicolle^14^14AIP NCP.11 pour les notes 13 à 17..

Ainsi : « Laveran, rapporteur de notre mémoire nous avait fait la … (mettez le mot que vous voudrez) de vouloir nous exclure; Roux s'est employé de son mieux à rattraper la chose; mais nous avons dû nous borner à un second prix. Vous comprendrez ainsi la raison de mes rapports avec Laveran. J'en ai eu pourtant pas mal d'officiels avec lui pour la constitution de cette Société de pathologie exotique dont vous serez et qui pourra, je crois, marcher^15^15Lettre de Félix Mesnil à Charles Nicolle du 16 décembre 1907. »

Laveran a 62 ans et vient de se voir décerner le prix Nobel; ses fonctions dans les Académies et la SPE l'amènent à prendre position pour des candidatures, des financements, des présentations de travaux scientifiques et son intégrité foncière, certains pencheraient plutôt pour une certaine rigidité, n'est pas toujours appréciée, au moins sur l'instant.

« Encore un merci cordial pour votre obligeance et mes regrets de n'avoir pas pu profiter de votre envoi de janvier, mais, vraiment, il n'y a pas de ma faute : Laveran prétendit (pas vous) que le chien était à nous deux, il l'a sacrifié en mai sans me faire signe. J'ai la prétention d'avoir de bonnes relations avec toutes les personnes qui s'y prêtent et je tiens à établir devant vous, mon ami, que les torts ne sont pas de mon côté. […] J'ai lu avec plaisir dans le *Temps* que Laveran vous avait fait donner un prix Montyon. Il n'est pas injuste avec tout le monde^16^16Lettre de Félix Mesnil à Charles Nicolle du 12 août 1909. »

Il est intéressant de noter que les échantillons biologiques voyageaient « simplement » à cette époque, soit envoyés, soit confiés à des personnes faisant le voyage. Là, ce sont des échantillons d'animaux vivants infectés par une leishmanie ou un trypanosome, à partir desquels le parasite pouvait être isolé et transmis à d'autres animaux sur le campus. Ce matériel biologique pouvait ainsi devenir source de friction, car important pour la poursuite de l'activité de recherche.

Lors du jubilé de Laveran, pour ses 70 ans, le 16 juin 1915 : « La cérémonie Laveran s'est bien passée. Roux a sorti ses souvenirs du Valde-Grâce et a dit fort gentiment au “Grand français” (bis) qu'il n’était pas aimable. L. paraissait enchanté; le lendemain il m'a fait les honneurs de ses souris à testicules et articulations enflés. C'est évidemment intéressant^17^17Lettre de Félix Mesnil à Charles Nicolle du 23 juin 1915.. »

Enfin, Charles Nicolle, apprenant le décès d'Alphonse Laveran, écrit, dans son style si particulier : « Mon cher ami, nos lettres se sont croisées comme le font, j'imagine, souvent nos pensées. C'est la seule ressource de l'amitié et la preuve de son effort lorsque la distance l'entrave. […] J'ai été peiné de la disparition de Laveran homme respectable et grincheux, toujours parfait pour moi; d'ailleurs excellent^18^18Lettre de Charles Nicolle à Louis Pasteur Vallery-Radot du 27 mai 1922, transcription transmise par Pierre Nicolle; AIP NCP.12.. » Ou comment adresser des piques tout en restant correct…

À côté de ces descriptions brèves de moments particuliers au laboratoire, Félix Mesnil évoque l'aspect « privé » d'Alphonse Laveran au cours de son allocution pour la cérémonie commémorative de sa découverte de l'hématozoaire du paludisme, le 23 mai 1930 à Constantine [14]:

« En dehors du laboratoire, à son foyer domestique, Laveran se révélait un homme différent. On s'apercevait de son goût pour la musique et les autres arts; on découvrait un fin connaisseur de notre littérature, un admirateur du Voltaire des *Contes* (il appréciait la philosophie de Candide), de l'Anatole France de l’*Histoire contemporaine*. Je me rappelle la période où « Monsieur Bergeret à Paris » paraissait dans le *Figaro* du mercredi; Laveran achetait régulièrement le numéro. » Anatole France commença à publier ces textes le mercredi 5 juillet 1899^19^19*Le Figaro*, 5 juillet 1899, https://gallica.bnf.fr/ark:/12148/bpt6k284807j., qui font partie de l’*Histoire contemporaine*, en quatre volumes, dont un se référant à l'affaire Dreyfus.

De même, Émile Marchoux (1862-1943) dans son discours lors de sa prise de fonction en tant que président de la SPE en 1928^20^20AIP LAV.6., écrira : « Une des plus grandes surprises qu’éprouvaient ceux qui avaient l'heur de lui plaire et de pénétrer dans son intimité, c’était de découvrir un homme tout différent de celui qu'ils connaissaient. Dans le particulier il devenait causeur, plaisant, gai, spirituel, attentif et charmant. »

Entre les adjectifs utilisés par Charles Nicolle – « respectable, grincheux, parfait, excellent » – et ceux d’Émile Marchoux – « causeur, plaisant, gai, spirituel, attentif, charmant », il y a un monde… Il est bien évident que ces citations ne fournissent qu'une vue partielle de la personne « Alphonse Laveran ». Nous avons tenté de proposer ce que nous avons ressenti à la lecture des différentes archives. Il est nécessaire d'opérer une synthèse la plus large possible à partir des écrits de ses contemporains pour tenter de cerner plus fidèlement le personnage.

Par exemple, en parcourant les feuilles de notes d'Alphonse Laveran au cours de ses différentes affectations, il est souvent mentionné dans la rubrique « Langues étrangères » qu'il maîtrisait l'allemand et l'italien, et une fois l'anglais^21^21SHD (Service historique de la Défense Vincennes) GR 9M 601 nos 92 & 94 par exemple.. Ceci est un atout important dans les échanges scientifiques, en particulier dans ce domaine des protozoaires dont l’étude est d'autant plus critique que les différentes sociétés, française, anglaise, allemande, italienne se sont lancées dans la colonisation de territoires où ces parasites sont endémiques, voire épidémiques, et vont donc grever lourdement la santé des « colons » militaires ou civils. De plus, en se remémorant la controverse Pasteur-Koch au congrès de Genève dont l'une des sources reposait sur la difficulté de compréhension du français par Robert Koch, tout autant que de l'absence de compréhension de l'allemand par Pasteur [23], cette fluidité linguistique permettait d’établir des connexions utiles pour le développement des idées et des concepts.

Enfin, une autre piste de recherche consiste à parcourir la presse quotidienne de l’époque, au cas où Alphonse Laveran s'y trouverait mentionné. Une seule occurrence (pour l'instant) fut retrouvée dans le *Figaro* du 6 janvier 1900, soit 20 ans après sa découverte et 7 ans avant son Nobel.

On peut ainsi lire : « Très jolie chambrée, avanthier, chez le docteur et Mme Barthélemy, dans leurs salons de la rue du Débarcadère. Le chansonnier Fursy s'est fait applaudir dans ses oeuvres. Dans l'assistance : L'explorateur Janne de Lamare, Mme Degalle, M. et Mme Marqueste, Mme de Fels, le professeur Laveran, etc. »

Le chansonnier Fursy, de son vrai nom Henri Dreyfus, est un chansonnier montmartrois de la Belle Époque; il venait de racheter, en 1899, le cabaret du Chat Noir. Janne de Lamare de l'Automobile Club de France effectua la traversée du Klondike en voiturette tricycle à moteur à pétrole en 1899. Qui est le docteur Barthélémy de la rue du Débarcadère dans le 17^e^ arrondissement, non loin de la Porte Maillot ? Le problème est qu'il s'agit d'un nom assez courant. Certaines pistes se présentent, mais nécessitent plus de recherches pour espérer trouver si c'est une connaissance d'Alphonse Laveran…

## LE CARNET DE CITATIONS D'ALPHONSE LAVERAN^22^22AIP LAV.4. (Fig. 4)

**Figure 4:**
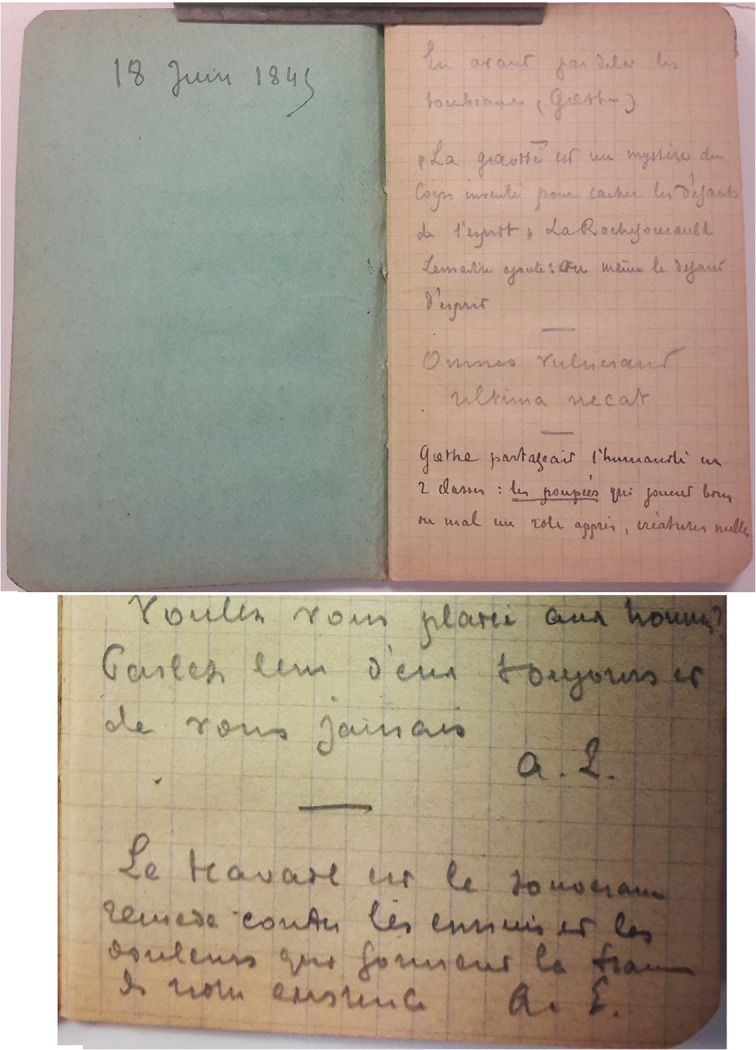
Carnet de citations d'Alphonse Laveran. Vue de la première page du carnet (en haut); exemple de citations signées A. L (crédit photo : Institut Pasteur/Archives - Fonds Alphonse Laveran) Alphonse Laveran's notebook of quotations. View of the first page of the notebook (top); example of quotations signed A. L (photo credit: Institut Pasteur/Archives - Fonds Alphonse Laveran)

Comment approcher autrement Alphonse Laveran ? En consultant son « carnet de citations »… En effet, dans les archives déposées à l'Institut Pasteur se trouve un petit carnet de 7,5 x 12 cm à petits carreaux, avec la date du 18 juin 1845 ajoutée manuellement au verso de la page de couverture, i.e. son année de naissance. Y figurent une trentaine de citations écrites de la main d'Alphonse Laveran. Elles nous donnent accès à une certaine vision de ses centres d'intérêt; certaines sont des phrases/aphorismes glanées au cours de ses lectures d'auteurs qu'il appréciait particulièrement; phrases qui sont entrées en résonance avec des moments particuliers de sa vie.

Nous n'avons aucune idée du moment où ces citations ont été notées; l'ont-elles été chronologiquement ? Ou bien recopiées au propre à une date ultérieure ? Ce qui est évident, c'est qu'elles recouvrent différents domaines et que ceux-ci sont fort divers. La recherche des dates de certains ouvrages cités avec les numéros de page et de tome pourrait nous fournir quelques précisions supplémentaires. Certaines sont des citations directement extraites des ouvrages, d'autres des reprises par d'autres auteurs, voire des mélanges… Les domaines couverts par ces citations peuvent se regrouper - arbitrairement (le privilège de l'interprétant…) - en plusieurs groupes. Seuls certains seront exposés ici pour ne pas trop allonger le propos. Une communication ultérieure fera découvrir les détails de ce document passionnant qui sont d'une actualité frappante.

Sur les « **amis** »:

« Les amis sont des gens à qui on est utile. Vieil [sic] Castel, *Mémoires,* t. II. p. 193^23^23Horace Viel-Castel (1802-1864), *Mémoires sur le règne de Napoléon III,* en date du 25 juin 1853 (https://archive.org/stream/mmoiresducomteh01vielgoog/mmoiresducomteh-01vielgoog_djvu.tx). ».

« Je suis charmée de faire de nouvelles connaissances, disait Mme de Staal Delaunay à Mme du Deffand, j'espère toujours qu'elles vaudront mieux que les anciennes. »

Il se reflète une certaine méfiance, voire une certaine réticence, vis-à-vis de la qualité des relations avec les personnes de son entourage…

Sur les « **apparences** »:

« La gravité est un mystère du corps inventé pour cacher les défauts de l'esprit », La Rochefoucauld. Lemaitre ajoute : « ou même le défaut d'esprit^24^24Jules Lemaitre (1853-1914) est un écrivain et critique dramatique français. Cette citation se trouve dans Impressions de théâtre, 10^e^ série de 1898 à propos de la comédie d'Abel Hermant, *La Carrière* (https://gallica.bnf.fr/ark:/12148/bpt6k2043462#, p. 125).. »

« Ce qui manque aux orateurs en profondeur, ils vous le donnent en longueur. Montesquieu, p. 135. »

De même, il ressort une certaine défiance vis-à-vis du paraître, d'une certaine superficialité qu'il devait rencontrer régulièrement autour de lui, dans le monde scientifique et médical, que ce fût dans les domaines civil ou militaire, dans ses souvenirs ou dans le réel. Ceci peut être mis en lien avec les souvenirs d’Émile Roubaud, Président de la SPE de 1936 à 1946 [20]:

« Les séances présidées par Laveran demeuraient strictement restreintes à l'exposé des communications. L’ère des causeries, illustrées de projections variées, qui fut inaugurée par Calmette dont nous avons fidèlement maintenu la tradition, n'avait pas encore vu le jour. Laveran, d'ailleurs, ne s'y fût guère prêté. On se bornait à présenter succinctement les notes et à les discuter rapidement. Lorsque le sujet lui en paraissait valoir la peine, le Président ajoutait à l'exposé quelques brèves réflexions… et c’était à peu près tout ce que l'on se permettait.

Son abord un peu froid et sévère, le sentiment d'austérité qui semblait s'attacher à sa personne imposaient d'ailleurs aux orateurs une réserve qui n’était guère propice à l'animation des séances. Un avantage pourtant, que vous me permettrez de souligner en passant, sans commentaires, c'est que ceux qui avaient la parole n'osaient pas beaucoup en abuser; aussi les séances y gagnaient-elles de ne jamais traîner en longueur. »

Autant le début de ce souvenir soulève le point de l’évolution inéluctable des savoirs et des faires, et la réticence de tout un chacun à les accepter, autant la fin illustre parfaitement le propos d'Alphonse Laveran et ne peut, même pour nous au xxi^e^ siècle, nous empêcher de reconnaître leur caractère encore contemporain lors de la gestion des temps de parole des orateurs…

Ou **de manière plus générale**:

« Il ne faut pas en vouloir à la vérité d’être parfois révolutionnaire; ce n'est pas sa faute : le vrai est ce qu'il peut (Goethe, convers. d'Eckermann t. 1, p. 310. »

Johann Peter Eckermann (1792-1854) avait recueilli ses entretiens avec Goethe à la fin de sa vie. Cette réflexion fait écho aux difficultés qu'Alphonse Laveran a rencontrées pour faire reconnaître sa découverte par la communauté scientifique et médicale… et donc son rapport à la vérité face aux opinions tranchées (retranchées ?) de ses collègues.

« Tout homme qui s’élève s'isole. » (Rivarol)

« Les moutons s'attroupent et les lions s'isolent. » (Rivarol)

Antoine Rivaroli, dit Rivarol (1753-1801), est un écrivain, journaliste, essayiste et pamphlétaire disciple de Voltaire. C'est l'auteur le plus cité (6 occurrences) dans ce carnet.

Cette notion d'isolement dans son entourage, tant militaire que scientifique, médical et civil, reflète cette sensation d'incompréhension de la part de ses pairs; de même que la vision de (devoir ?) penser par soi-même et non selon les conceptions « acceptées », les présupposés de l’époque, conduit immanquablement à l'isolement en s'excluant des concepts « académiques » du moment.

Cela exige de « ne pas avoir la nostalgie d’être comme tout le monde » comme le formule si élégamment Isabelle Stengers^25^25Peut-on décoloniser les sciences ? Autour de la question, RFI, émission du 7 juin 2022..

Enfin, certaines citations particulières portent la signature « A. L. » (Fig. 4). Ce qui suggère très fortement que ce sont des réflexions personnelles qu'il a considérées suffisamment importantes au moment de leur éclosion pour être transcrites dans son carnet et les conserver. Les voici:

« Nous sommes tous des condamnés à mort. Bienheureux quand la nature ne nous applique pas la torture avant de nous tuer. A. L. » Nous n'avons pas d'idée de la date à laquelle cette réflexion a été transcrite, bien sombre il est vrai. Est-ce la disparition des personnes qui lui étaient chères autour de lui, ou la sienne propre, entrant fortement en résonance avec sa fin de vie, à un moment où il se savait condamné ?

« Voulez-vous plaire aux hommes ? Parlezleur d'eux toujours et de vous jamais. A. L. »

Ce qui suggère bien le manque de confiance et le désabusement face à ses contemporains et rejoint les citations sur les amis mentionnées précédemment.

Et enfin:

« Le travail est le nouveau remède contre les ennuis et les douleurs qui forment la trame de notre existence. A. L. »

… Une certaine vision de la vie, de sa vie en particulier ? Il faut en effet se souvenir de ce qu'Alphonse Laveran a vécu depuis jeune adulte. À 25 ans, au sortir de sa formation de médecin militaire à Strasbourg, il a connu le siège de Metz pendant la guerre francoprussienne de 1870, puis la commune de Paris en 1871. Dans ses affectations en Algérie, il est confronté quotidiennement à la mort des patients civils ou militaires, en particulier de la malaria. Il se trouve à Constantine lors du décès de son père – et y reste –, sa consolation pourrait-on imaginer est de consacrer une grande partie de son temps au microscope à observer inlassablement, l'esprit ouvert.

Il est soumis aux critiques, parfois virulentes (il est intéressant de lire les débats à fleurets mouchetés à l'Académie nationale de médecine) sur sa découverte du parasite du paludisme. Les relations exécrables avec sa hiérarchie militaire dans les années 1894-1896 aboutissent à sa démission. Bref, la sensation de solitude, d'isolement, de sa propre différence par rapport aux autres a dû le marquer profondément…

Est également survenu un évènement de « fronde étudiante » pendant ses études à l’École impériale du Service de santé militaire de Strasbourg (1863-1867), mentionné de façon fort sibylline dans un courrier de Jean-Louis Rouis à Louis Théodore Laveran du 13 juillet 1864^26^26MSSA C1043_dos3.. Cette année-là, le casernement obligatoire avait été décidé pour tous les élèves (auparavant répartis entre divers logements en ville du fait du manque de capacité d'hébergement) et le recrutement d’élèves pharmaciens avait été mis en place [10, 21]. Le casernement n'avait pas été bien perçu, étant ressenti comme une diminution de liberté, aboutissant à des mouvements d'agitation/rébellion dès 1864 et culminant en juillet 1866.

Nous ne savons pas si Alphonse Laveran, alors âgé de 19-21 ans, fut entraîné, même de loin, dans ces mouvements de contestation. Dans une autre lettre du 19 juillet 1867^27^27MSSA C1043_dos3., Rouis mentionne : « Dans quelques jours, Mr votre fils touchera au terme de sa scolarité. Je suis heureux de pouvoir vous apprendre que depuis Pâques nous n'avons eu aucune observation à lui adresser au point de vue de la discipline. » Cette formulation ambiguë pourrait suggérer que, avant Pâques… ?

Il est tentant de se demander s'il y eut participation et quel retentissement « officieux » aurait pu exister.

Enfin, la lecture des lettres envoyées par Charles Emmanuel Sédillot, directeur de l’École impériale du Service de santé militaire de Strasbourg, Jean-Louis Rouis, sous-directeur, Charles Henri Ehrmann, doyen de la Faculté de médecine, au père d'Alphonse Laveran à propos du déroulement des études de son fils montrent également une pression non négligeable sur le suivi et les résultats demandés^28^28MSSA C1043_dos3..

Toutes ces réflexions amènent à considérer que l'isolement, la concentration solitaire d'Alphonse Laveran autour de son microscope et ses observations aient pu être favorisés. Ces citations nous donnent un aperçu d'Alphonse Laveran. Il pensait par lui-même et ne voulait pas dépendre des pensées et considérations des autres, donc « académiques » (dans le sens péjoratif du terme); il était ainsi ouvert à sa propre vision, fût-elle en désaccord avec les connaissances et présupposés de l’époque.

Cela pouvait amener certains conflits dans les discussions, même si parfois il s'avérait que sa conception pouvait être possiblement, probablement, erronée, et il lui fallait du temps pour intégrer et digérer les données nouvelles dans sa vision personnelle et ainsi réajuster son modèle. Ainsi, Émile Roubaud écrit en 1946 [20] : « Très entier dans ses conceptions, Laveran est, en effet, jusqu’à la fin de sa vie demeuré étroitement attaché à celles qu'il avait formulées, en dépit des contradictions possibles. […] Mais s'il n'acceptait point, en apparence, ces données d'une expérience, aujourd'hui classique, qui s'opposaient à sa thèse bien connue sur *l'unicité,* dans le fond de lui-même, il en était demeuré certainement frappé. Et j'en eus plus tard la preuve en feuilletant les fiches bibliographiques qu'il m'avait léguées. Sur celle qui a trait à mon mémoire de 1918, sur la transmission du paludisme par les anophèles des régions non palustres, on peut lire, sans commentaires ni discussions, cette phrase qui lui était apparue comme résumant le fond de la question : “L’évolution du *Pl. vivax* est un peu plus rapide que celle du *Pl. præcox* !” »

L’être humain préfère en effet la zone de confort du déjà connu, et c'est tout à son honneur d'accepter de s'en éloigner et de découvrir ainsi de nouveaux horizons. Le délai pour se décentrer est en lien étroit avec le malaise ressenti par les deux parties en confrontation; plus il est court, plus la relation scientifique devient enrichissante et ouvre la porte à des surprises et des étonnements, moteur de notre activité de recherche et d'enseignement. Pour ceux d'entre nous qui avons été amenés à faire accepter une conception, une vision singulière, ou seulement des données inattendues, cela ne peut que nous parler.

## ALPHONSE LAVERAN ET SON ÉGARD POUR LES PERSONNES QU'IL CÔTOIE DANS LA VIE DE TOUS LES JOURS

**Figure 5:**
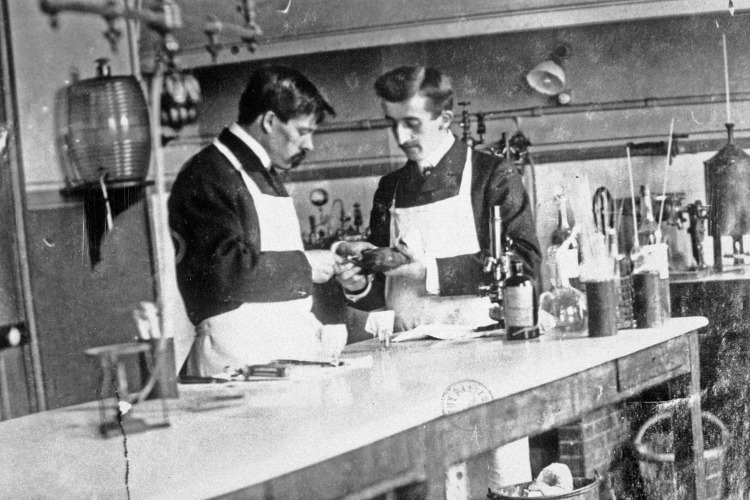
Léon Breton vers 1890-1900. Léon Breton (à droite) et Adrien Latapie dans le laboratoire d'Élie Metchnikoff vers 1890-1900 (crédit photo : Institut Pasteur/Musée Pasteur) Léon Breton around 1890-1900. Léon Breton (right) and Adrien Latapie in Élie Metchnikoff's laboratory around 1890-1900 (photo credit: Institut Pasteur/Musée Pasteur)

Alphonse Laveran est attentif aux personnes avec lesquelles il interagit tous les jours, que ce soit au laboratoire ou à son domicile. Ceci est explicite à la lecture de ses dispositions testamentaires en date du 29 septembre 1921 concernant les legs à sa cuisinière et à son garçon de laboratoire^29^29AN MC/ET/L/1797. Ainsi : « Je lègue la somme de 1 000 Fr à Mr Léon Breton mon 1^er^ garçon de laboratoire qui m'a toujours aidé dans mes travaux avec beaucoup de zèle. » Léon Breton était assistant dans le service de Metchnikoff depuis 1890 (Fig. 5); son adresse mentionnée dans l'acte notarié de délivrance de legs (daté des 5 octobre et 7 novembre 1922) est le 96 rue Falguière à Paris, représentant le bâtiment « Laveran » sur le site de l'Institut Pasteur et il est dénommé « aide préparateur de laboratoire ». Le salaire d'un aide préparateur varie entre 2 400 (3^e^ classe) et 3 600 Fr (1^re^ classe) par an, tel que mentionné lors du Conseil d'administration de l'Institut Pasteur du 19 janvier 1910 abordant la hiérarchie et le traitement du personnel^30^30IP LAV.2.. Ce legs représentait donc au moins trois mois de salaire pour Léon Breton.

Sur le versant personnel, dans son testament : « Je lègue à notre cuisinière Mlle Annette Badillet une somme de 3 000 Fr. Si Mlle Badillet reste au service de Me Laveran ma femme jusqu’à la mort de celle-ci, je prie Me Laveran de lui léguer une rente dont le total devra représenter 100 Fr de rente par année de service, en reconnaissance du dévouement qu'elle nous témoigne depuis plus de 20 ans. » Dans un codicille ajouté le 30 septembre 1921, il ajoute : « Si ma femme vient à mourir avant moi et dans ce cas seulement, je lègue à Mlle Annette Badillet, si elle est encore à mon service au jour de mon décès, une rente annuelle et viagère, dont le total devra représenter 100 Fr de rente par année de service (Mlle A. Badillet est entrée à notre service le 6 octobre 1900). Je répète que ce legs ne peut avoir d'effet que si ma femme est décédée avant moi. Fait et écrit en entier de ma main. À Paris le 30 septembre 1921. »

Ce legs est délivré en octobre/novembre 1922, Marie Laveran signe « M. Laveran née Pidancet »^31^31AN MC/ET/L/NC/126, 127, 130 et 201.. À son décès le 27 janvier 1950, Marie Laveran lègue 50 000 Fr à sa cuisinière Amélie Badillet^32^32AP DQ7 30520.. Est-ce la même personne ? Annette ? Amélie ? Dans cette succession se trouve ajouté de façon manuscrite qu'elle serait née le 13 novembre 1870 (?) dans le Loir-et-Cher. Si cette personne est bien la même, elle serait entrée au service du couple Laveran à l’âge de 30 ans et serait restée chez eux pendant 50 ans, âgée de 80 ans au moment du décès de Mme Laveran.

Ces documents nous permettent ainsi d’éclairer certaines facettes de la personne d'Alphonse Laveran.

### Les personnalités féminines autour de Laveran

À une époque où les contributions et l'influence des femmes dans la vie de la société commencent enfin à être dévoilées et reconnues après une période d'effacement actif par certains membres et groupes de cette société, il nous semble important d'apporter notre contribution à ce pan de vie d'Alphonse Laveran.

#### Marie Guénard de la Tour, sa mère

La première mention d'Alphonse Laveran retrouvée dans les archives le décrit comme enfant, à Metz. Son père y fut affecté de 1847 à 1851, Alphonse Laveran approchait probablement de ses 6 ans au vu des évènements décrits dans ce témoignage de Charles François Alexandre Perron déjà cité précédemment. Suite à une invitation à déjeuner par Louis Théodore Laveran, « Quoique un peu [sic] intimidé d'abord, je me sentis bientôt presque à l'aise dans ce milieu d'honnêteté, avec une maîtresse de maison douce et prévenante et des enfants qu'on n'entendait pas. C’était un intérieur charmant. »

Le calme des enfants à table est aussi le reflet de l’éducation qui était prodiguée au xix^e^ siècle et de leur place dans le milieu familial. Se trouvent ainsi décrites deux personnes qui jouèrent un rôle non négligeable dans la vie d'Alphonse Laveran : sa mère et sa sœur Caroline.

Alphonse Laveran dédicace à sa mère sa Thèse de médecine en 1867 (« À ma bonne mère. Souvenir affectueux »), et plus tard son ouvrage *Contribution à l’étude de la tuberculose aiguë* en 1873 (« Hommage à ma chère maman »)^33^33MSSA C1043_dos5 et C1043-1_dos5 respectivement..

À son retour d'Algérie en 1884, après « La Découverte » en 1880, Alphonse Laveran est nommé professeur au Val-de-Grâce pour 10 ans. Il rentre donc à Paris et loge au 58 rue Denfert dans un bâtiment de 5 étages construit en 1853. Cette rue a été rebaptisée depuis, car elle a été interrompue par le percement du boulevard Saint Michel. Elle est devenue la rue Henri Barbusse pour les numéros jusqu’à 55 et 64, et l'avenue Denfert

Rochereau pour les numéros au-delà de 59 et 68, les noms ont changé mais les numéros ont été conservés… Ce domicile (actuellement 58 rue Henri Barbusse) déclaré dans son précontrat de mariage du 11 août 1885^34^34SHD GR 9M 601 n° 66-72. se trouve juste à côté du Val-de-Grâce; comme son père, il loge près de son lieu de travail qu'il peut gagner à pied. Il est intéressant de noter que sa mère, veuve depuis 1879, est mentionnée habitant au 25 de la rue Denfert, donc pas loin de chez lui (obligations mentionnées dans l'acte notarié, cession de valeurs du 25 juillet 1922) ^35^35AN MC/ET/L/NC/126, 127, 130 et 201.; elle a donc quitté l'appartement du 127 boulevard Saint Michel où elle vivait avec son mari Louis Théodore Laveran et sa fille Caroline.

#### Caroline Laveran, sa sœur

Au travers des actes notariés, se dessine le portrait d'une femme qui a vécu avec ses parents jusqu'au décès de son père (127 boulevard Saint Michel), dont elle a assuré la succession en l'absence de son frère, puis avec sa mère (25 rue (ancienne) Denfert Rochereau, puis 7 avenue du Maine) jusqu’à son propre décès en 1929 à l’âge de 87 ans à la même adresse. Elle s'est ainsi occupée de ses deux parents. À partir d'un certain moment, elle a souffert d'une cécité; ceci est mentionné dans le testament d'Alphonse Laveran en 1921 et visible dans sa signature d'un acte notarié de cessions de valeurs en faveur de Marie Laveran veuve d'Alphonse Laveran en date du 25 juillet 1922 (cf. note 33). Dans cet acte très probablement rendu nécessaire pour pallier le retentissement de son handicap sur la vie quotidienne, Marie Laveran a en charge le versement d'une rente annuelle viagère de 9 845 Fr en quatre paiements. Elle utilise comme signature « M. Pidancet Laveran ».

Maintenant tournons-nous vers deux personnalités plus particulièrement, qui ont interagi avec Alphonse Laveran au cours de sa vie et ont joué un rôle considérable pour « perpétuer » sa mémoire; deux femmes, Marie Laveran, son épouse, et Marie Phisalix, une collègue et biographe (voire hagiographe ?).

#### Sophie Marie Pidancet, son épouse

**Figure 6:**
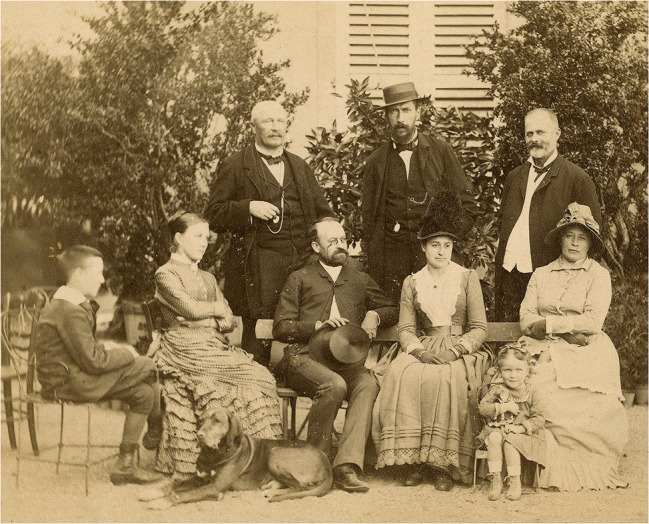
Alphonse Laveran entouré d'amis à l'époque de son mariage. Alphonse Laveran, assis avec son chapeau haut de forme à côté de sa future épouse Sophie Marie Pidancet, dans le jardin chez M. Mathieu à Novéant-sur-Moselle (crédit photo : Institut Pasteur/Musée Pasteur) Alphonse Laveran surrounded by friends at the time of his marriage. Alphonse Laveran, seated with his top hat next to his future wife Sophie Marie Pidancet, in the garden of Mr Mathieu's home in Novéant-sur-Moselle (photo credit: Institut Pasteur/Musée Pasteur)

Le 5 octobre 1885, à l’âge de 45 ans, Alphonse Laveran épouse Sophie Marie Pidancet âgée de 27 ans à Montoy-Flanville à la périphérie de Metz. Metz se trouve alors dans les zones annexées par la Prusse, ce qui fait que dans les tables décennales ils se trouvent tous deux nommés « Karl Alfons Ludwig » et « Sophia Maria » (Fig. 6)…

Alphonse Laveran doit demander à ses autorités hiérarchiques une autorisation de mariage, ainsi que cela était obligatoire à l’époque^36^36Cette obligation fut supprimée par le général Louis André (1838-1913) *(Cinq ans de ministère,* 1909, pp. 81-91, https://gallica.bnf.fr/ark:/12148/bpt6k54505714).. Deux documents sont alors nécessaires, l'un témoignant de la « bonne réputation » de la future épouse, l'autre s'assurant des moyens de subsistance du futur ménage. Ainsi trouvet-on dans les archives une lettre adressée par le maire de Montoy-Flanville en date du 7 août 1885^37^37SHD GR 9M 601 n° 73. : « Nous maire de la Commune de Montoy (Alsace Lorraine) confirmons qu'il résulte des renseignements exacts que nous nous sommes procurés que Mademoiselle Marie Pidancet âgée de 27 ans fille de Mr Louis Pidancet et de Mme Pidancet née Clesex, demandée en mariage par Mr le Docteur A. Laveran Médecin Principal, Professeur au Val-de-Grâce jouit d'une bonne réputation ainsi que sa famille, qu'elle aura en mariage sept mille francs de rente et que des espérances de fortune peuvent être évaluées à environ cent mille francs. »

**Figure 7:**
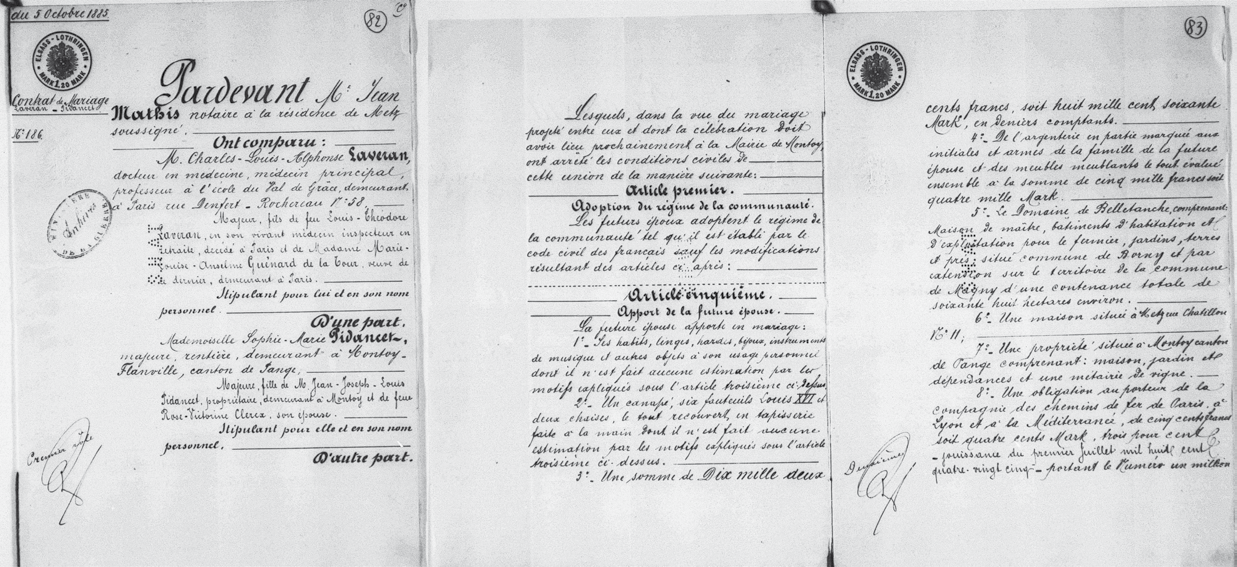
Contrat de mariage entre Alphonse Laveran et Sophie Marie Pidancet. Premières pages du contrat établi le 5 octobre 1885 (crédit photo : Service historique de la Défense/Vincennes) Marriage contract between Alphonse Laveran and Sophie Marie Pidancet. First pages of the contract drawn up on 5 October 1885 (photo credit: Service historique de la Défense/Vincennes)

Dans le précontrat et le contrat de mariage sont ainsi mentionnés environ 17 000 Fr en biens et obligations, un domaine de 68 ha, une maison à Metz, une propriété viticole, 10 000 Fr de créances, 4 572 Fr de revenus et 2 400 Fr d'arrérages de fermage^38^38SHD GR 9M 601 n^os^ 82 à 87. (Fig. 7). Ce mariage est typique des accords matrimoniaux de l’époque : d'après Gilbert Percebois [17], cette union a été arrangée *via* une connaissance (et ami ?) qui se trouve témoin à ce mariage, « le médecin major de 1^re^ classe des Sapeurs-pompiers de la ville de Paris, Marie Gustave Léon Régnier, 42 ans, demeurant à Paris »; il est effectivement mentionné comme témoin dans l'acte de mariage de la mairie de Montoy-Flanville^39^39SHD GR 9M 601 n° 88.. Où et dans quelles circonstances Alphonse Laveran a-t-il connu Régnier et comment cette présentation se fit-elle ? Ils étaient tous deux au siège de Metz [17]; sinon restent les « trous » de l'histoire… qui peuvent ouvrir la porte à l'imagination…

Sophie Marie Pidancet provient donc d'une famille aisée et va donc apporter à Alphonse Laveran une source de revenus non négligeable en complément de son revenu de médecin militaire et de ses propres ressources héritées lors du décès de son père^40^40AP DQ7 11403. On peut imaginer que cette source financière matrimoniale lui sera d'une grande aide, et peutêtre un argument pour décider de remettre sa démission en 1897.

En effet Marie Phisalix, dans sa biographie d'Alphonse Laveran, mentionne le caractère bénévole de sa position à l'Institut Pasteur : « Il alla porter sa renommée et son travail, qu'il voulut dès lors bénévole, à l'Institut Pasteur où MM. Duclaux et Roux lui firent un très bon accueil. Il devint chef de service honoraire. »

Alphonse Laveran ne pouvait évidemment pas savoir qu'il recevrait le prix Nobel en 1907 (environ 190 000 Fr, mentionné dans la lettre du 31 octobre du recteur du Karolinska Institutet A. H. Mörner^41^41MSSA C1043_dos14. [3]).

**Figure 8:**
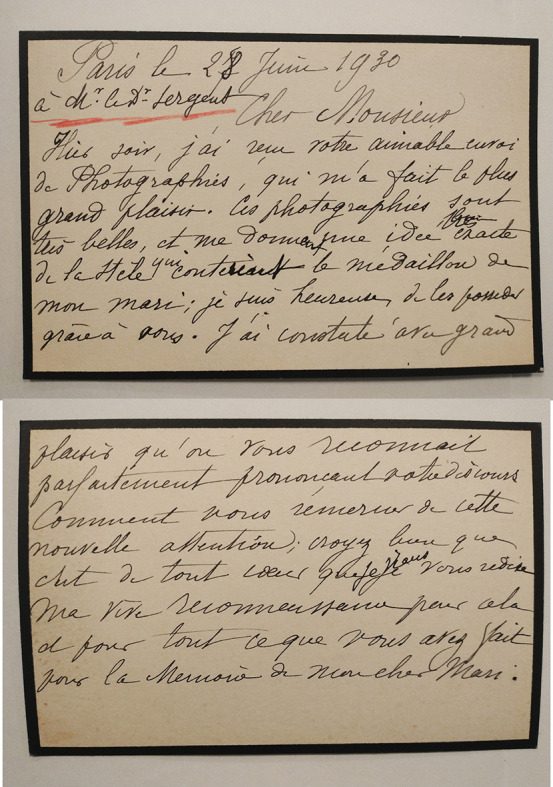
Brouillon/copie de lettre de Marie Laveran à Edmond Sergent en date du 28 juin 1930 à l'occasion des cérémonies à Alger et Constantine pour le cinquantenaire de La Découverte (crédit photo : Institut Pasteur/Archives – Fonds Alphonse Laveran) Draft/copy of a letter from Marie Laveran to Edmond Sergent dated 28 June 1930 on the occasion of the ceremonies in Algiers and Constantine to mark the fiftieth anniversary of La Découverte (photo credit: Institut Pasteur/Archives - Fonds Alphonse Laveran)

Marie Laveran, née Sophie Marie Pidancet, vivra avec lui jusqu’à son décès. Elle jouera un rôle important après le décès de son mari. Elle représente l'interlocutrice des scientifiques qui désirent rendre hommage à Alphonse Laveran. Ceci ressort de la lecture de ses courriers conservés aux archives de l'Institut Pasteur^42^42AIP LAV.5.. Les moyens de reproduction n'existaient pas à l’époque, à part la copie manuelle, ce que Marie Laveran faisait sur des feuilles bordées de noir, en mentionnant quel était le destinataire et en conservant les enveloppes (un exemple en Fig. 8). Ces échanges épistolaires montrent une intimité entre les différents scientifiques et leurs familles; les femmes, enfants sont souvent cités et les bonnes et mauvaises nouvelles partagées^43^43Par exemple dans les courriers avec Félix Mesnil, Charles Nicolle, Émile Marchoux, Edmond Sergent, Albert Calmette….

À titre d'exemple, il est intéressant de lire les échanges lors de la préparation et de la tenue du congrès sur la « découverte de Laveran à Constantine » à Alger en mai 1930, pour son cinquantenaire, sachant que cela coïncidait avec le centenaire de la « conquête de l'Algérie » par la France. En voici une sélection:

Le 28 mars 1930, Marie Laveran répond à Edmond Sergent:

« J'espérais pouvoir répondre à votre aimable invitation, et me rendre en Algérie, pour les fêtes du cinquantenaire de la découverte de mon mari, que vous avez organisées, et pour lesquelles vous vous êtes dépensé avec tant de dévouement.

Mais je me sens trop fatiguée, trop âgée [72 ans], et dans un état de santé qui ne me permet pas d'entreprendre ce voyage. »

Émile Marchoux envoie un télégramme le 19 mai 1930, depuis Alger à Mme Laveran:

« Tous congressistes acclamants [sic] nom Laveran envoient hommages respectueux à compagne grand savant. »

Et Félix Mesnil depuis Constantine le 22 mai:

« Congressistes envoient de Constantine respectueux hommages. »

À la suite du congrès, Marie Laveran écrit de Paris le 28 juin 1930 à Edmond Sergent:

« Hier soir, j'ai reçu votre aimable envoi de photographies, qui m'a fait le plus grand plaisir. Ces photographies sont très belles et me donnent une idée (très/bien) exacte de la stèle qui contient le médaillon de mon mari; je suis heureuse de les posséder grâce à vous. […] croyez bien que c'est de tout cœur que je vous redise ma vive reconnaissance pour cela et pour tout ce que vous avez fait pour la mémoire de mon cher mari. »

Et le 25 août 1931:

« J'ai reçu ce matin le volume du compte-rendu du Congrès du paludisme, qui s'est tenu à Alger, l'année dernière. […] Je viens donc vous exprimer toute ma reconnaissance qui s'ajoute à la vive gratitude que je vous dois déjà pour tant de choses faites à la mémoire de mon mari.

J'espère que vous, et toute votre Famille vous êtes en bonne santé. Votre charmante Fille [sic] est aimablement venue me voir l'hiver dernier; comme elle ne recevait plus à ce moment, je n'ai pu aller la remercier. Dès que la saison des visites recommencera je m'empresserai d'aller la voir. »

Ce courrier montre non seulement les échanges et les liens entre les différents acteurs de cette histoire scientifique^44^44Félix Mesnil, Émile Marchoux, Edmond Sergent, Albert Calmette…, mais également nous donne un aperçu de la vie en société à cette époque, où la saison des visites représentait une norme sociale à respecter.

Marie Laveran faisait également preuve d'un certain sens de l'humour… Ainsi, recevant un courrier dactylographié d'un certain Virgilio A. Planas de Rosario de Santa Fe en Argentine avec une photo jointe de Laveran, demandant à Alphonse Laveran d'apposer sa signature sur celle-ci en vue d'une collection d'autographes de gens de lettres argentins et étrangers (!), Marie Laveran inscrit sur l'enveloppe de cette missive : « Répondu – que le Professeur Laveran est mort le 18 mai 1922 ». Le courrier datait du 23 février 1928^45^45AIP LAV.7. !

#### Marie Félicité Picot, épouse Phisalix (Fig. 9)

Marie Laveran, à travers ses courriers, présente une personnalité attachante; nous en verrons un peu plus dans sa correspondance avec une « scientifique-médecin » dont le nom est lié à celui de Laveran, Marie Phisalix, née Marie Félicité Picot (1861-1946), originaire de Besançon.

**Figure 9:**
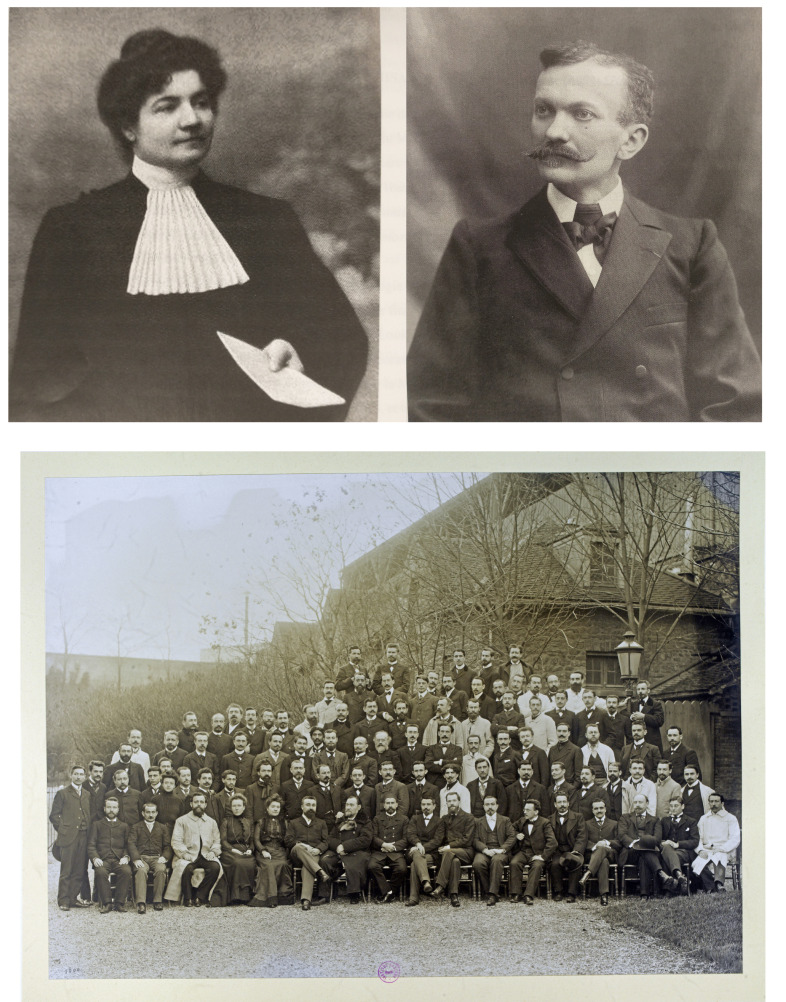
Marie Félicité Picot et son mari Césaire Phisalix. Marie Phisalix, docteure en médecine en 1900, suit le cours de microbie technique de l'Institut Pasteur en 1900; elle est assise au premier rang (5^e^), Metchnikoff, Roux et Borrel des 7^e^ aux 9^e^ positions (crédit photo : Institut Pasteur/Musée Pasteur) Marie Félicité Picot and her husband Césaire Phisalix. Marie Phisalix, doctor of medicine in 1900, attended the technical microbiology course at the Institut Pasteur in 1900; she is seated in the front row (5^th^), with Metchnikoff, Roux and Borrel in the 7th to 9^th^ positions (photo credit: Institut Pasteur/Musée Pasteur)

Elle sort diplômée de l’École normale supérieure des jeunes filles de Sèvres en 1882 (cf. le numéro 124 du *Bulletin de la Société Herpétologique de France* qui lui est consacré en 2007), obtient son agrégation de sciences en 1888 et soutient en 1900 une Thèse de médecine intitulée « Recherches histologiques, embryologiques et physiologiques sur les glandes à venin de la salamandre terrestre », récompensée par la médaille d'argent de la Faculté de Paris, figurant ainsi parmi les premières femmes médecin en France [5]. En 1900, elle suit le cours de microbie technique de l'Institut Pasteur (Fig. 9), étant l'une des trois femmes présentes sur le cliché de la promotion; Alphonse Laveran a rejoint l'Institut Pasteur 3 ans auparavant en 1897.

L'un des aspects clefs de la vie de Marie Phisalix est sa rencontre avec Césaire Phisalix (Fig. 9) et leur mariage en 1895. Césaire Phisalix est médecin militaire, élève d'Alphonse Laveran en 1876-1877. Pour des raisons de santé (entérocolite fiévreuse acquise lors de la campagne de Tunisie en 1881, fièvre typhoïde supposée^46^46SHD GR 5Yf 55809. ?), il prend sa retraite de l'armée en 1887 et entre au Muséum national d'Histoire naturelle (MNHN) en 1888. Il y mène des recherches sur les venins de serpent et découvre avec Gabriel Bertrand l'immunothérapie passive (ou transfert passif de l'immunité humorale), aboutissant à son article du 10 février 1894 « Recherches expérimentales sur le venin de vipère – Propriétés antitoxiques du sang des animaux vaccinés contre le venin de vipère ». Césaire Phisalix ne s'est jamais remis de sa pathologie intestinale et décède en 1906. Gabriel Bertrand, son collaborateur au MNHN, ayant été « repéré » par Émile Duclaux qui avait succédé à Pasteur à la tête de l'Institut, rejoindra l'Institut Pasteur ultérieurement (1900); le monde scientifique est petit…

Marie Phisalix était entrée au MNHN en 1895, date de son mariage avec Césaire Phisalix, en tant qu’« attachée bénévole au MNHN (et restera dans cet emploi jusqu’à sa mort) [9] »; elle est mentionnée comme « héritière d'une fortune confortable », lui procurant aisance financière et indépendance [11]. Elle continue l’œuvre de son mari après son décès, devenant ainsi ce que la Société d'herpétologie de France a nommé « une grande dame de l'Herpétologie [11] »; elle intègre le laboratoire de Zoologie (Reptiles et poissons) avec sa spécialité des venins, de la pathologie animale, dont les serpents en particulier, menant ses recherches sur les amphibiens et les reptiles. Marie Phisalix effectue toute sa carrière au MNHN, spécialiste reconnue internationalement des venins de serpent et assimilés. Elle rédige ainsi une somme en deux tomes, 1500 pages environ en tout, abondamment illustrée, intitulée *Animaux venimeux et venins*^47^47www.biodiversitylibrary.org/bibliography/11934; ou sur archive.org.. Cet ouvrage de référence a la particularité entre autres d’être préfacé par Alphonse Laveran lui-même, le 17 octobre 1921; date à laquelle il se savait déjà condamné (il commença à rédiger son testament le 29 septembre); il s'y réfère comme « membre de l'Institut et de l'Académie de médecine ». Cet ouvrage est publié en 1922; rappelons-nous qu'Alphonse Laveran décède le 18 mai 1922.

## GENESE DE L'ŒUVRE PRINCEPS *ALPHONSE LAVERAN, SA VIE, SON ŒUVRE* PAR MARIE PHISALIX (Fig. 10)

**Figure 10:**
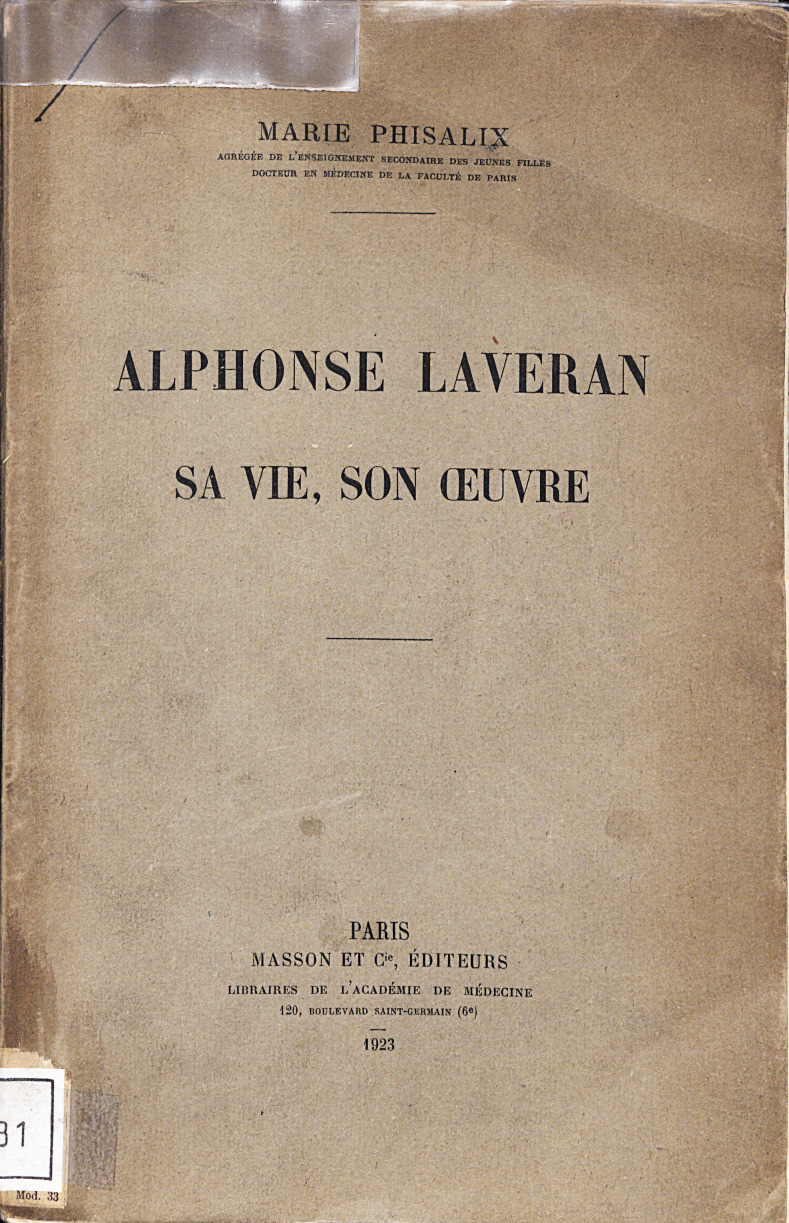
Page de garde du livre de Marie Phisalix sur Alphonse Laveran, publié en 1923 (crédit photo : Institut Pasteur/Archives – Fonds Alphonse Laveran). Accessible sur la plateforme PaJ@Mo du CeRIS à l'Institut Pasteur, https://bibnum.pasteur.fr/app/photopro.sk/pasteur/doclist?fpsearch=laveran+phisalix#sessionhistory-1688030012069 *Cover page of Marie Phisalix's book on Alphonse Laveran, published in 1923 (photo credit: Institut Pasteur/Archives - Fonds Alphonse Laveran). Accessible on the CeRIS PaJ@Mo platform at the Institut Pasteur,* https://bibnum.pasteur.fr/app/photopro.sk/pasteur/doclist?fpsearch=laveran+phisalix#session-history-1688030012069

Marie Phisalix publie début 1923 une œuvre princeps, la seule biographie disponible d'Alphonse Laveran, *Alphonse Laveran, sa vie, son œuvre,* aux éditions Masson. Cette biographie est mentionnée par Félix Mesnil dans le discours officiel qu'il fit à Constantine le 23 mai 1930, lors de la « cérémonie commémorative de la découverte par Alphonse Laveran de l'hématozoaire du paludisme [14] » : « On trouvera aussi un résumé de son œuvre dans le livre que Mme Phisalix lui a consacré l'année qui a suivi sa mort; c'est le seul monument qu'ait voulu Laveran pour perpétuer sa mémoire. »

Cette œuvre est devenue iconique pour les études sur Alphonse Laveran, la majorité d'entre nous nous y référons, car elle est exhaustive, admirablement écrite et illustrée, et elle aborde tous les aspects des activités multiples et protéiformes d'Alphonse Laveran. Elle est souvent citée expressément, parfois et plus rarement juste utilisée sans référence; mais tout lecteur de cet ouvrage reconnaît immanquablement l'origine des informations utilisées…

Ainsi, et cela donnera une vision très claire de l'humour et de la causticité de Marie Phisalix, le 10 décembre 1929, Charles Achard (secrétaire général de l'Académie de médecine et membre de l'Académie des sciences) prononce l’éloge d'Alphonse Laveran à l'Académie de médecine [1]. Dans une lettre du 24 novembre 1929 à Marie Laveran (invitée à cette cérémonie)^48^48AIP LAV.5., Marie Phisalix écrit : « Chère Madame Amie, […] en lui répondant, pour pour [sic] préciser à Achard, demandez-lui de vous réserver 3 places à côté l'une de l'autre dont une pour Mme Phisalix, car il aura tiré de ma notice les éléments techniques de son discours, puisqu'il ne vous a rien demandé de plus. »

Et Marie Laveran de répondre à Charles Achard le lendemain^49^49AIP LAV.5. : « Je vous remercie de votre lettre m'annonçant qu’à la séance publique annuelle de l'Académie de médecine vous prononcerez l’éloge de mon mari.

Je serai heureuse de me rendre à votre invitation et d'assister à cette séance. […] Je vous serais mille fois reconnaissante de bien vouloir me réserver 3 places voisines, afin que je puisse avoir à côté de moi deux dames qui m'accompagneront, et dont l'une sera Mme Phisalix. » Dans la feuille récapitulant les cartes demandées par Marie Laveran pour cette séance, la troisième personne invitée est « Annette », sa cuisinière comme mentionnée dans les testaments d'Alphonse Laveran et de Marie Laveran.

Abordons maintenant la genèse de cette « notice » d'après les documents présents dans les archives, ce qui éclairera d'une lumière nouvelle ce document clef. Dans une lettre du 23 février 1923 de Félix Mesnil à Charles Nicolle, le livre est déjà paru : « Le bouquin Laveran-Phésa.. [sic dans la transcription] a paru. Vous recevrez d'ici peu 3 exemplaires (vous, Burnet, votre bibliothèque). Mme Laveran m'ayant dit de ne pas vous oublier, vous pourrez lui accuser réception^50^50AIP NCP.11.. »

**Figure 11:**
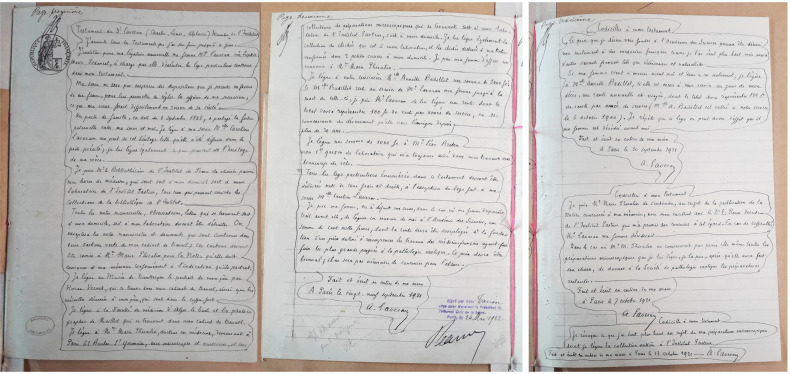
Testament olographe d'Alphonse Laveran. Archives nationales, MC/ET/L/1797 Holograph will of Alphonse Laveran. Archives nationales, MC/ET/L/1797

Or, pour ceux d'entre nous qui ont eu à assurer la rédaction et la publication d'un ouvrage, même si l'on en est le seul auteur, le délai entre le décès d'Alphonse Laveran le 18 mai 1922 et la parution d'un tel hommage/contribution posthume semble fort court. La lecture du testament d'Alphonse Laveran (Fig. 11)^51^51AN MC/ET/L/1797. donne les clefs de ceci.

Le 29 septembre 1921, Alphonse Laveran écrit dans son testament : « Toutes les notes manuscrites, observations, lettres qui se trouvent soit à mon domicile, soit à mon laboratoire devront être détruites. On exceptera les notes manuscrites et documents qui sont contenus dans deux cartons verts de mon cabinet de travail; ces cartons devront être remis à Mme Marie Phisalix pour la Notice qu'elle doit consacrer à ma mémoire conformément à l'indication qu'ils portent. »

Et un peu plus loin : « Je lui lègue également la collection de clichés qui est à mon laboratoire, et les clichés destinés à ma Notice renfermés dans 2 petites caisses à mon domicile. » Pour conclure, Alphonse Laveran rajoute le 7 octobre 1921 : « Codicille à mon testament. Je prie Mme Marie Phisalix de s'entendre, au sujet de la publication de la Notice consacrée à ma mémoire, avec mon excellent ami le Dr É. Roux directeur de l'Institut Pasteur qui m'a promis son concours à cet égard. »

Enfin, dans une lettre datée du 30 octobre 1922, soit 5 mois et demi après le décès d'Alphonse Laveran, Félix Mesnil écrit à Charles Nicolle^52^52AIP NCP.11.:

« Nous faisons imprimer 400 pages sur Laveran ! Tranches de son œuvre que Mme Phi.. [sic dans la transcription] a soudée sous l’œil attentif du Maître. Il n'y a personne sans vanité, mais elle se place diversement. » Il ressort donc qu'Alphonse Laveran a demandé à Marie Phisalix d’écrire cette « Notice » avec les documents et clichés qu'il lui fournissait et sous sa supervision. Il avait sûrement une immense confiance en ses capacités de scientifique et d’écrivaine pour mettre en forme cette (auto?) biographie à un moment de sa vie où il voyait s'approcher l'issue finale. Nous pourrions ainsi considérer que cette « Notice » comme il la nomme dans son testament, avec un N majuscule, est son testament scientifique, fournissant à la postérité les aspects de sa vie qu'il souhaitait laisser. Un contrôle absolu de son image… Effectivement, la mention dans son testament de la destruction de tous ses papiers montre bien cette volonté de ne laisser que ce qu'il avait décidé devoir rester. Exigence testamentaire qui fut *a priori* scrupuleusement suivie comme en témoigne cet extrait de la lettre de Marie Phisalix du 24 novembre 1929, soit 7 ans après son décès^53^53AIP LAV.7. : « Les lettres sont débrouillées, j'ai déchiré celles qui ne signifient pas grand-chose et qui émanaient de contemporains quelconques; de cette façon les désirs de Mr Laveran sont en partie réalisés. Les autres documents vont suivre, […]. »

Poursuivons sur Marie Phisalix : ainsi que l'on a pu le lire plus haut, elle n'a pas « sa langue dans la poche ». Charles Achard en a fait les frais… et, de même, Émile Roux, directeur de l'Institut Pasteur, se fait gentiment « chambrer »… Dans une lettre du 8 décembre 1930 à Marie Laveran, elle écrit : « Chère Madame Amie, […] Enfin M. Roux s'est aussi décidé; car après tout il ne pouvait pas ne pas dire merci de la photo de sa promotion sur laquelle – a-t-il dit – “il avait plus de cheveux qu'aujourd'hui”. On ne saurait tout avoir; le calotin pastorien est une sérieuse compensation^54^54AIP LAV.5.. »

Dans sa lettre du 24 novembre 1929 à Marie Laveran, Marie Phisalix écrit : « J'ai trouvé le nom également de l'américain de Baltimore enthousiaste de la France et des français; c'est Thayer. Ainsi qui cherche trouve; il est vrai qu'on trouve quelquefois autre chose que ce que l'on cherche^55^55AIP LAV.7.… » Une philosophie de l'activité scientifique qu'elle vit tous les jours et une observation fort sensée et indémodable…

Marie Phisalix était bienvenue au domicile de Marie Laveran. Le 14 novembre 1929, Marie Phisalix lui écrit : « Chère Madame Amie, le jeudi 21 nov. me convient parfaitement, et j'accepte votre bonne proposition de déjeuner, vers midi, à 15 m. près, car on ne peut maintenant compter que sur le train 4 [?] et le métro^56^56AIP LAV.5.. » Elle y avait son « jour »; ainsi dans sa lettre du 8 décembre 1930^57^57AN MC/ET/L/1797. : « Je ne reprendrai pas mon jour avant janvier, car il m'est tombé toutes espèces de tuiles dans mon casuel, entre autres choses des caisses de serpents d'Amérique du Sud, qu'il me faut employer aussitôt pour ne pas perdre ce gibier coûteux et rare. […] À bientôt, et bonne amitié. M. Phisalix »

Elle n'a pu assister aux cérémonies du centenaire de la naissance de Laveran le 12 juillet 1945, juste après la fin de la Seconde Guerre mondiale; elle avait commencé à avoir des problèmes de santé ainsi qu’Émile Roubaud le mentionne dans son discours à la Société de pathologie exotique le 9 janvier 1946 [20] : « Par infortune aussi, Mme Phisalix, sur qui nous comptions plus spécialement pour évoquer cette grande figure à qui elle a consacré un ouvrage particulièrement attachant, se trouve encore sous le coup d'une affection sérieuse, qui l'astreint à garder la chambre. En lui adressant tous nos vœux de prompt et d'entier rétablissement, je vais tenter de la remplacer, si possible, auprès de vous. »

Marie Phisalix décède à Paris le 19 janvier 1946 à 23 heures, âgée de 84 ans, au 151 rue de Sèvres; cette adresse est celle de l'hôpital Necker et le déclarant sur l'acte de décès ne connaît ni sa profession ni les noms de père et mère. Elle est inhumée au cimetière de Bagneux le 23 janvier 1946, et transférée le 5 novembre 1947 seulement auprès de son mari à Mouthier-Haute-Pierre (village natal de son mari) dans le Doubs près de Besançon^58^58AP n° 1752 dans le registre d'inhumation du cimetière de Bagneux. où elle avait créé un musée d'histoire naturelle en 1907 après le décès de Césaire Phisalix, musée qui porte son nom^59^59www.cancoillotte.net/spip.php?article512 et https://racinescomtoises.net/index?/category/4220-marie_phisalix_1861_1946..

Curieusement, Marie Phisalix et Alphonse Laveran n'ont co-publié qu'une seule fois en 1913 (M. Phisalix et A. Laveran, Sur une hémogrégarine nouvelle de *Lachesis alternatus,* Bull Soc Pathol Exot, 1913, t. VI, n° 5). Mais, sans être exhaustif, en ne relevant que certaines publications mentionnées dans le livre de Marie Phisalix, on s'aperçoit qu'Alphonse Laveran a publié dès 1890 sur les hématozoaires de nombreux animaux : d'oiseaux, dès 1901-1902, hémogrégarines de poissons (avec Mesnil), d'ophidiens, trypanosomes de poissons et de grenouille (toujours avec Mesnil), de requin en 1908 avec la description d'une hémogrégarine et d'un trypanosome, etc. pour ne donner qu'un aspect des domaines de recherche sur ces parasites, nouveaux pathogènes en pleine découverte. Au vu de ces publications d'Alphonse Laveran sur les parasites en particulier de serpents, il est stimulant de se poser la question de l'existence possible de liens avec le MNHN, et donc Césaire puis Marie Phisalix, dans le recrutement de ces échantillons. Rappelons que Césaire Phisalix est entré au MNHN en 1888 et y a travaillé jusqu’à sa mort en 1906 sur les venins de serpent. Marie Phisalix y est entrée en 1895, année de son mariage et y a accompli toute sa carrière.

## Laveran et la Maitrise de son Image, une Iconologie (Fig. 12)

**Figure 12:**
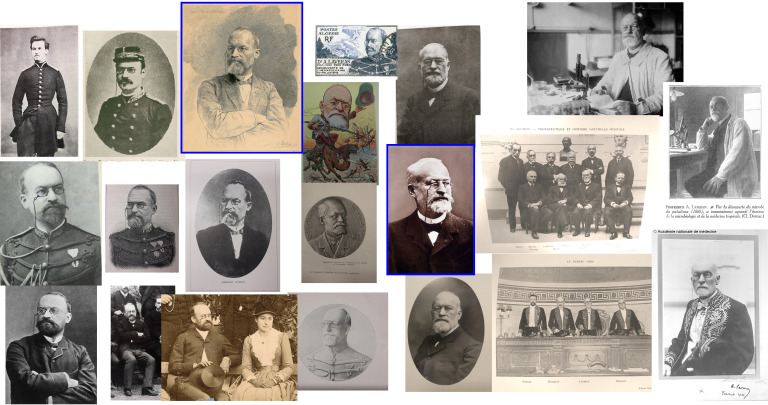
Montage iconographique d'Alphonse Laveran dont, encadrés, le dessin gravé par Émile Boilvin en 1894 (en haut) et le portrait par Eugène Pirou vers 1902-1904 (au milieu) (crédit photo : Institut Pasteur/Musée Pasteur) Montage of images of Alphonse Laveran including, framed, the engraved drawing by Émile Boilvin in 1894 (top) and the portrait by Eugène Pirou circa 1902-1904 (middle) (photo credit: Institut Pasteur/Musée Pasteur)

Alphonse Laveran a maîtrisé son image, tant scientifique qu'humaine. Il ne semble pas exister pléthore de photographies ou gravures ou caricatures d'Alphonse Laveran; il est vrai que l’ère des selfies et des photos immédiates n’était pas encore advenue. On retrouve ainsi quelques photos de sa période militaire, jeune étudiant, ou plus tard dans son uniforme, avec son lorgnon, moustache, puis barbe « bouc » sombre, puis grisonnante, lobes frontaux bien dégagés. On peut l'admirer en costume d'académicien, en blouse dans son laboratoire auprès de son cher microscope…

Cependant, deux représentations émergent, d'après une lettre du 2 juillet 1926 de Marie Laveran à Monsieur Prudhomme qui devait exécuter un médaillon à l'effigie d'Alphonse Laveran soit 4 ans après le décès de son mari^60^60AIP LAV.5. : « Vous recevrez 2 photographies et la gravure du regretté Émile Boivin [sic]. Ce beau portrait a été exécuté en 1894 d'après nature, mon mari avait alors 49 ans, la ressemblance était parfaite.

Ce que je vois depuis pouvant le plus vous être utile, c'est la photo contenue dans la brochure *(Alphonse Laveran, sa vie, son œuvre,* par Mme le Dr Marie Phisalix). Cette photo, de Pirou, également très ressemblante, date de 1902 ou 1904, c'est celle qui a servi lorsque mon mari a eu le prix Nobel, c'est le plus répandu de ses portraits, celui qui est le plus connu à l’étranger. Il avait alors environ 60 ans. Les dimensions de la tête, et la forme du visage sont également bien indiquées dans les deux portraits. Je crois que c'est dans ces 2 images que vous pourrez trouver le mieux les indications [indicateurs ?] qui vous seront nécessaires.

J'ai joint (parce qu'elle donne le profil) la petite charge que le médecin inspecteur général Delorme s'est amusé à faire pendant une séance de l'Académie de médecine. Le crâne est trop volumineux, mais dans le nez, la bouche, tout le bas du visage, il y a quelque chose à retenir. »

**Figure 13:**
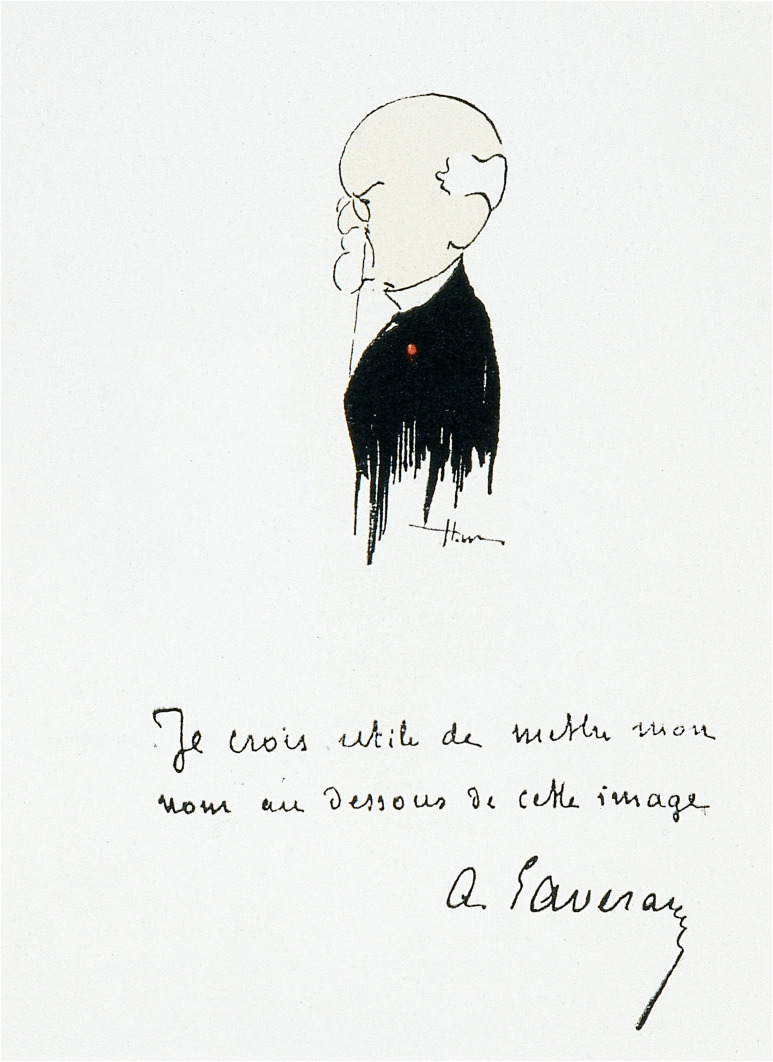
Portrait caricature d'Alphonse Laveran (1845-1922) fait par le médecin inspecteur général Delorme pendant une séance de l'Académie de médecine (cf. lettre de Marie Laveran du 2 juillet 1926, AIP LAV.5). In *Album de l'Académie de médecine,* Laboratoires Fournier, Pl. XI, 1910 (crédit photo : Institut Pasteur/Musée Pasteur) Caricature portrait of Alphonse Laveran (1845-1922) drawn by Inspector General Delorme during a session of the Académie de médecine (cf. letter from Marie Laveran dated 2 July 1926, AIP LAV.5). In Album de l'Académie de médecine, Laboratoires Fournier, Pl. XI, 1910 (photo credit: Institut Pasteur/Musée Pasteur)

Faisant toujours preuve d'un humour caustique, même pour lui-même, Alphonse Laveran inscrit en dessous de son portrait à charge à l'Académie de médecine : « Je crois utile de mettre mon nom au dessous [sic] de cette image^61^61MSSA C1043_1 dos12. » (Fig. 13). Clairement, ce qu'Alphonse Laveran souhaitait laisser était l’œuvre à laquelle il a consacré sa vie.

**Figure 14:**
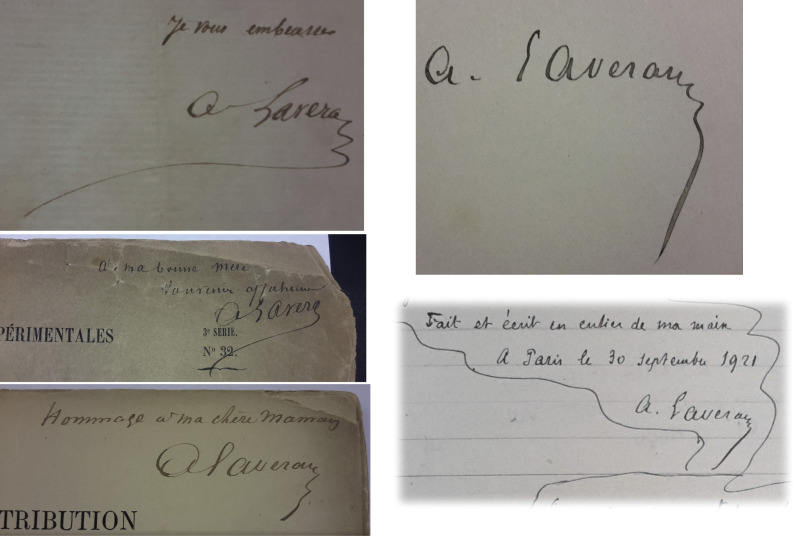
Exemples de signatures d'Alphonse Laveran. De haut en bas et de gauche à droite : lettre d'Alphonse Laveran à sa mère lui annonçant sa réussite aux examens finaux de l'École impériale du Service de santé à Strasbourg en 1867 (« Je suis reçu avec le n° 1 », MSSA C1043_dos4); dédicaces à sa mère de sa Thèse de médecine en 1867 (MSSA C1043_dos5) et de son ouvrage sur la tuberculose aiguë en 1873 (C1043-1_dos5); signature « classique » stabilisée (sans date, vers 1907-1910 ?, AIP DR.CR.2); signature sur le testament olographe en septembre/octobre 1921 (Archives nationales, MC/ET/L/1797) Examples of Alphonse Laveran's signatures. From top to bottom and left to right: Alphonse Laveran's letter to his mother announcing his success in the final examinations at the École impériale du Service de santé in Strasbourg in 1867 (“Je suis reçu avec le n° 1", MSSA C1043_dos4); dedications to his mother of his Thèse de médecine in 1867 (MSSA C1043_dos5) and of his work on acute tuberculosis in 1873 (C1043-1_dos5); stabilised “classical” signature (undated, circa 1907-1910? AIP DR.CR.2); signature on holograph will in September/October 1921 (Archives nationales, MC/ET/L/1797)

Sa signature, par contre, il ne l'a pas censurée ! avec le trait descendant de plus en plus bas à la fin du « n » caractéristique^62^62AIP LAV et MSSA C1043 & C1043_1 (documents divers). (Fig. 14), retrouvé déjà dans la dédicace de sa thèse à sa mère en 1867, ou en 1873 pour son travail sur la tuberculose aiguë, et déjà modifié par rapport à la lettre par laquelle il informe sa mère de son succès à Strasbourg^63^63MSSA C1043_dos5, C1043-1_dos5 et dos4 respectivement.. signatures fort différentes de celle de son père^64^64MSSA C1042.. Il est amusant de noter que cette jambe du « n » poursuivant son mouvement vers le bas se retrouve aussi dans la signature de Marie Laveran dans un acte notarié en juillet 1922^65^65AN MC/ET/L/NC/126, 127, 130 et 201..

La signature, après une longue gestation du vi^e^ au xvi^e^ siècle, est devenue progressivement un témoignage d'identité caractérisant le signataire et sa singularité, une marque personnelle, parfois considérée comme une trace, une empreinte du corps et de l’âme^66^66Elle est également signe de validation d'un document, le transformant ainsi en lui conférant une authenticité (documents diplomatiques par exemple, mais que nous côtoyons tous les jours dans nos démarches officielles). [7]. Que pourrions-nous imaginer et déduire sur Alphonse Laveran ?…

## Commentaires De Conclusion

Pour conclure, du moins provisoirement, nos recherches nous permettent de faire entendre les voix des contemporains d'Alphonse Laveran, vivant entre la fin du xix^e^ et le début du xx^e^ siècle, et d'approcher Alphonse Laveran au sein de cette société et dans le cadre de son activité de recherche.

Nous ne sommes pas les premiers à évoquer la singularité d'Alphonse Laveran dans le monde scientifique, au milieu de ses pairs, personnalité aussi rugueuse que singulière. Émile Roux et Émile Roubaud l'avaient déjà évoqué. Laissons-leur la parole…

Émile Roux:

« Si, au lieu de vous concentrer dans un travail solitaire, vous aviez poursuivi vos recherches dans un laboratoire fréquenté, vous auriez pu être entraîné hors de votre propre sentier et vous engager dans la voie où tout le monde se jetait, mais qui ne conduisait pas où vous vouliez aller [22]. »

Émile Roubaud:

« À l'heure où il nous est tant parlé de recherche dirigée, d'esprit d’équipe, de travail en série pour aboutir à ce rendement scientifique intensif que l'on nous présente en idéal, cette phrase d’É. Roux doit être particulièrement méditée [20]. »

Cette dernière réflexion date d'il y a plus de 75 ans, mais elle est terriblement d'actualité… Il y a donc besoin d'une pluralité de modes de fonctionnement, non seulement pour l'activité scientifique, mais nous pourrions l’étendre aux approches de la vie et des travaux d'une personne historique, comme Alphonse Laveran.

En effet, d'un côté l'on peut parfaitement considérer que seuls les écrits scientifiques (au sens large) qui ont été publiés sont à conserver et éliminer tout le reste, le considérant superflu – cette approche fut celle suivie (involontairement) par les premiers directeurs de l'Institut Pasteur, aboutissant à la disparition d'un nombre d'archives de la première époque pasteurienne [16] qui, maintenant avec le recul, manquent tant aux historiens pour reconstituer cette période.

De l'autre, l'on peut écrire un récit d'une vie à partir des fragments d'archives qui nous sont parvenus. Comme le formule Hervé Mazurel reprenant les propos de Paul Ricœur:

« Une vie demeure avant tout un tissu d'histoires racontées en constante reconfiguration : l'histoire d'une vie ne cesse d’être refigurée par toutes les histoires véridiques ou fictives qu'un sujet raconte sur lui-même [13] ». Et l'on pourrait compléter : et refigurée aussi par les histoires écrites ultérieurement sur le sujet devenu alors objet.

Bien évidemment, une approche mixte est sans doute la plus riche, permettant d'analyser le devenir et les concepts de la science, la démarche scientifique, dans le contexte de la société et de la famille.

Enfin, que reste-t-il à écrire sur Alphonse Laveran ? Bien d'autres aspects pourraient être abordés, comme le mystère de ses microscopes…

Mais cette contribution s'est étirée sur un nombre déjà conséquent de pages pour ne pas s'attirer les foudres d'Alphonse Laveran tel qu'il l'a noté dans son carnet, en confondant profondeur et longueur… Cependant, quand on fait le bilan de la littérature (pléthorique ?) autour de Shakespeare et de Molière jusqu’à nos jours au vu de la paucité des documents primaires existants, nous pouvons nous réjouir des opportunités d'analyse et de découverte qui s'offrent à nous… Il existe encore des fonds documentaires non totalement explorés, voire inexplorés; une approche « décalée » peut également permettre de visualiser autrement ce qui est connu, su ou considéré comme tel. Cette dernière approche est ce qui s'apparente à une démarche scientifique enrichissante, car permettant de se « décentrer », se « déraciner », s’éloigner de l'attendu et s'ouvrir à l'inconnu qui peut surgir, émerger sans que l'on puisse le prédire, la création de nouveau à partir de l'ancien, l'ouverture à l'inattendu. Comme l'a dit Pasteur, « souvenez-vous que dans les champs de l'observation le hasard ne favorise que les esprits préparés^67^67Discours prononcé à Douai, le 7 décembre 1854, *Œuvres de Pasteur réunies par Pasteur Vallery-Radot,* tome VII).. »

## REMERCIEMENTS

Je souhaiterais vivement remercier toutes celles et ceux qui ont contribué au développement de cette approche, en priant celles et ceux que j'aurais pu oublier de m'en excuser : Catherine Cécilio, Clarice Celli, Christophe Cloquier, Marion Denise, Sandra Legout, Michèle Mock, Marc Morillon, Michèle Périssère, Annick Perrot, Chantal Pflieger, Denis Ringaud, François Rodhain, Christian Sany, Maxime Schwartz, Cédric Thépenier, Jean-Nicolas Tournier.

Ainsi que les structures:

Archives, Musée et Bibliothèque de l'Institut Pasteur (AIP), Archives, Musée et Bibliothèque du Service de santé des armées Valde-Grâce (MSSA), Archives du Service historique de la Défense Vincennes (SHD), Archives de Paris (AP), Archives nationales site de Paris (AN), Mairie de Montoy-Flanville.

Je souhaiterais particulièrement mentionner Aymé Camelin, Gilbert Percebois et André Dodin qui ont tenté bien avant moi d'approcher Alphonse Laveran de manière décentrée. Les moyens de recherche disponibles actuellement sont différents, en particulier l'explosion numérique de ces dernières années ont facilité bien des choses, mais l'esprit de curiosité est toujours le même.

## Liens D'intérêts

L'auteur ne déclare aucun lien d'intérêt.
